# DNA methylation age acceleration contributes to the development and prediction of non-alcoholic fatty liver disease

**DOI:** 10.1007/s11357-023-00903-5

**Published:** 2023-08-22

**Authors:** Mingfeng Xia, Wenran Li, Huandong Lin, Hailuan Zeng, Shuai Ma, Qi Wu, Hui Ma, Xiaoming Li, Baishen Pan, Jian Gao, Yu Hu, Yun Liu, Sijia Wang, Xin Gao

**Affiliations:** 1 Department of Endocrinology and Metabolism, Zhongshan Hospital, Fudan Institute for Metabolic Diseases, Fudan University, 180 Fenglin Rd, Shanghai, 200032 China; 2 Human Phenome Institute, Fudan University, Shanghai, 201203 China; 3 CAS Key Laboratory of Computational Biology, Shanghai Institute of Nutrition and Health, University of Chinese Academy of Sciences, Chinese Academy of Sciences, 320 Yueyang Rd, Shanghai, 200031 China; 4 Department of Geriatrics, Zhongshan Hospital, Fudan University, Shanghai, 200032 China; 5 Department of Laboratory Medicine, Zhongshan Hospital, Fudan University, Shanghai, 200032 China; 6 Department of Nutrition, Zhongshan Hospital of Fudan University, Shanghai, 200032 China; 7 Department of Biochemistry and Molecular Biology, School of Basic Medical Sciences and Zhongshan Hospital, Fudan University, Shanghai, China; 8 Center for Excellence in Animal Evolution and Genetics, Chinese Academy of Sciences, Kunming, 650223 China

**Keywords:** Non-alcoholic fatty liver disease, Horvath age, DNA methylation age acceleration, Body fat distribution

## Abstract

Non-alcoholic fatty liver disease (NAFLD) is prevalent in the aging society. Despite body weight reduction, the prevalence of NAFLD has been increasing with aging for unknown reasons. Here, we investigate the association of DNA methylation age acceleration, a hallmark of aging, with risk of NAFLD. Genome-wide DNA methylation profiles were measured in 95 participants who developed type 2 diabetes during 4-year follow-up, and 356 randomly sampled participants from Shanghai Changfeng Study. DNA methylation age was calculated using the Horvath’s method, and liver fat content (LFC) was measured using a quantitative ultrasound method. Subjects with highest tertile of DNA methylation age acceleration (≥ 9.5 years) had significantly higher LFC (7.2% vs 3.1%, *P* = 0.008) but lower body fat percentage (29.7% vs 33.0%, *P* = 0.032) than those with lowest tertile of DNA methylation age acceleration (< 4.0 years). After adjustment for age, sex, alcohol drinking, cigarette smoking, BMI, waist circumference, and different type blood cell counts, the risk of NAFLD was still significantly increased in the highest tertile group (OR, 4.55; 95% CI, 1.06–19.61). Even in subjects with similar LFC at baseline, DNA methylation age acceleration was associated with higher increase in LFC (4.0 ± 10.7% vs 0.9 ± 9.5%, *P* = 0.004) after a median of 4-year follow-up. Further analysis found that 6 CpGs of Horvath age predictors were associated with longitudinal changes in LFC after multivariate adjustment and located on genes that might lead to fat redistribution from peripheral adipose to liver. Combination of the key CpG methylation related to liver fat content with conventional risk factors improves the performance for NAFLD prediction.

**Graphical Abstract:**

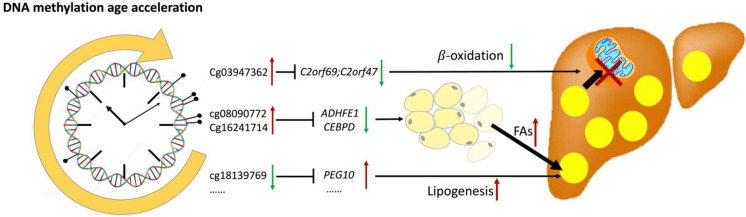

**Supplementary Information:**

The online version contains supplementary material available at 10.1007/s11357-023-00903-5.

## Introduction

Non-alcoholic fatty liver disease (NAFLD) is the leading cause of chronic liver disease that afflicts about 25% of the world’s population [1]. The histologic spectrum of NAFLD ranges from simple steatosis to non-alcoholic steatohepatitis (NASH), liver fibrosis, and eventually cirrhosis or hepatocellular carcinoma, and approximately 20–30% of patients with NAFLD can progress to NASH, a more severe form of NAFLD with liver inflammation and cellular damage. It has been recognized that NAFLD is a heterogeneous liver disease caused by dynamic interactions between both genetic and environmental factors [2]. Although NAFLD is usually associated with obesity, diabetes, metabolic syndrome, and cardiovascular disease [3], not all NAFLD patients are overweight or obese [4]. Susceptibility to NAFLD appears to accumulate with age. The prevalence of NAFLD continuously rises from 30–39 to 70–79 years old, despite a trend of body weight reduction after the age of 50 [5]. Almost half of octogenarians have NAFLD, but their prevalence of diabetes and metabolic syndrome is not higher than those without NAFLD [6]. The aged liver is characterized by progressive physiological alterations [7], and one hallmark of aging at the hepatocyte level is alterations in DNA methylation [8]. DNA demethylation could cause liver steatosis, inflammation, and fibrosis in mice fed a methyl-deficient diet [9]. In humans, aged liver was featured of differential methylation including but not limited to the regions associated with the WNT pathway and epithelial-to-mesenchymal transition according to previous studies [10].

Recently, a predictor of biological age based on DNA methylation (“Horvath clock”) was established [11]. Different from chronological age, methylation age could be modified by the environmental factors. Obesity accelerates tissue aging measured by “Horvath clock” [12], while lifestyle weight loss intervention could reverse the methylation aging [13]. One recent study found that DNA methylation age acceleration was observed among the patients with NASH [14]. However, few studies have investigated the relationship and mechanism between DNA methylation age acceleration and incidence of NAFLD longitudinally.

In the current study, we determined DNA methylation age in a cohort of 95 participants with new-onset type 2 diabetes during 4-year follow-up and a randomly sampled cohort of 356 participants from Shanghai Changfeng Study. We investigated the association between DNA methylation age acceleration and risk of NAFLD in these two representative cohorts. The key CpG sites related to NAFLD were identified, and their effects on gene regulation and lipid metabolism were further discussed.

## Materials and methods

### Study subjects

Our study analyzed two population cohorts from Shanghai Changfeng Study, a community-based prospective cohort study of chronic diseases among middle-aged and elderly residents from Shanghai Changfeng Community [15]. The inclusion criteria were age 45 years or older, and the exclusion criteria included refusal to participate in the study or refusal to sign an informed consent form. Initially, genome-wide DNA methylation profiles were measured in the whole blood of 100 participants with new-onset type 2 diabetes at follow-up examination (Cohort 1) as described previously [16] and a randomly sampled representative cohort of 407 participants (Cohort 2), who were selected using SPSS software by a computer. After excluding the participants with missing liver ultrasound examination, excessive alcohol consumption, or other known chronic liver diseases, 95 participants from Cohort 1 and 356 participants from Cohort 2 were included for further analysis. The study was approved by the Research Ethics Committee of Zhongshan Hospital, Fudan University (Nos. 2008–119 and B2013-132). Written informed consent was obtained from each participant.

### Anthropometric and biochemical measurements

The past history of alcohol drinking, cigarette smoking, chronic diseases, and medication use of each participant was collected in a face-to-face interview with a trained investigator using a standardized questionnaire. The questionnaire is modified from the questionnaire of the Rotterdam Study according to the lifestyle and customs of people in Shanghai [15]. Height and weight were measured without shoes or outer clothing. Body mass index (BMI) was calculated as the weight in kilograms divided by the square of height in meters (kg/m^2^). Fat masses at the whole body, trunk, and limbs sites were measured by a single, trained technician using dual-energy X-ray absorptiometry (Lunar iDXA, GE Healthcare). Resting systolic blood pressure (SBP) and diastolic blood pressure (DBP) were measured using an electronic blood pressure monitor (OMRON Model HEM-752 FUZZY, Omron Co., Dalian, China). Blood samples were collected after a 12-h overnight fasting. Serum biochemical parameters, including serum alanine transaminase (ALT), aspartate transaminase (AST), fasting plasma glucose (FPG), oral glucose tolerance test (OGTT) 2-h post-load plasma glucose (PPG), total cholesterol (TC), triglycerides (TG), and high-density lipoprotein (HDL) cholesterol, were measured using an automated bio-analyzer (HITACHI 7600, Tokyo, Japan). Low-density lipoprotein (LDL) cholesterol was calculated using the Friedewald equation.

### Ultrasound quantification of liver fat content (LFC)

Hepatic ultrasound examination was performed using a GE LOGIQ P5 ultrasound machine (GE Healthcare, Milwaukee, WI) with a 4-MHz abdominal probe. Since the degree of liver steatosis is positively correlated with the echogenicity of liver in comparison to that of renal cortex and ultrasound beam attenuation under ultrasonography, we quantified the ultrasound hepatic/renal ratio and hepatic attenuation rate using a computer program and estimated LFC using these two parameters. As detailed in our previous work [17], ultrasound images of liver/right kidney sagittal view and right liver lobe intercostal view were captured under the ultrasound machine and analyzed using a NIH image software (ImageJ 1.41o, National Institutes of Health, Bethesda, MD). To avoid the interference of intrahepatic blood vessels and bile ducts, representative regions of interest (ROI) in the liver anterior margin (depth 4–6 cm), liver posterior margin, and the liver parenchyma-right renal cortex at the same depth were selected at homogeneous areas, and the echo intensity within the ROIs was measured. We also measured the linear distance between the ROIs at the liver anterior margin and posterior margin. The ultrasound hepatic/renal ratio and hepatic attenuation rate were calculated using the following equations derived from the ultrasound exponential attenuation law as follows:

$$\mathrm{Ultrasound\;hepatic}/\mathrm{renal\;ratio}=\mathrm{ultrasound\;echo\;intensity\;in\;the\;liver\;ROI}/\mathrm{ultrasound\;echo\;intensity\;in\;the\;renal\;cortex\;ROI}$$

$$\mathrm{Ultrasound\;hepatic\;attenuation\;rate}=\left({\mathrm{lnA}}_{\mathrm{n}}-{\mathrm{lnA}}_{\mathrm{f}}\right)/{\left(\Delta d\times f\right)}^{17}$$

where *A*_n_ and *A*_f_ are the average ultrasound echo intensity in the liver near-field and far-field ROIs, respectively, ⊿*d* is the distance between the above two ROIs, and *f* represented the frequency of the ultrasound detector.

Both ultrasound hepatic/renal ratio and hepatic attenuation rate were standardized by the measured hepatic/renal ratio and hepatic attenuation rate of the tissue mimicking phantom under the same machine setting condition, and the LFC was then calculated as LFC (%) = 62.592 × standardized ultrasound hepatic/renal ratio + 168.076 × standardized ultrasound hepatic attenuation rate − 27.863. The LFC determined by the quantitative ultrasound method had an excellent correlation with that obtained by proton magnetic resonance (*r* = 0.89, *P* < 0.001), and the LFC cutoff for fatty liver by the ultrasound quantitative method was 9.15%, with a sensitivity of 95.1% and specificity of 100%, according to our previous study [17].

### Definitions

Fatty liver is identified when liver fat content by ultrasonography exceeded the cutoff value of 9.15% [17]. NAFLD is diagnosed with evidence of hepatic steatosis as determined by liver ultrasonography and exclusion of excessive alcohol consumption, or self-reported history of other chronic liver diseases, medication use, or other reasons that may cause hepatic steatosis. Metabolic associated fatty liver disease (MAFLD) is defined as hepatic steatosis diagnosed by liver ultrasonography in the presence of at least one of the following three metabolic conditions: overweight/obesity, type 2 diabetes, or at least two of the seven metabolic risk abnormalities as detailed previously [18]. Incidence of NAFLD/MAFLD was defined if NAFLD/MAFLD could not be diagnosed at baseline but confirmed during the follow-up. The criteria for metabolic syndrome were central obesity (waist circumference ≥ 90 cm for men and ≥ 80 cm for women) plus two or more of the following components: (1) TG levels ≥ 1.7 mmol/L or receiving drug treatment for elevated triglycerides, (2) HDL cholesterol levels < 1.03 mmol/L in men or < 1.29 mmol/L in women or receiving drug treatment for reduced HDL cholesterol, (3) SBP/ DBP ≥ 130/85 mmHg or antihypertensive drug treatment in a patient with a history of hypertension, and (4) FPG ≥ 5.6 mmol/L, or drug treatment for elevated glucose, according to the International Diabetes Federation criteria [19]. Hypertension was defined as SBP/DBP ≥ 140/90 mmHg or antihypertensive drug treatment in a patient with a history of hypertension. Hypertriglyceridemia was defined as a TG ≥ 2.3 mmol/L or a previous history of hypertriglyceridemia or drug treatment for elevated triglycerides, and hypercholesterolemia was defined as a TC ≥ 5.2 mmol/L and/or LDL-C ≥ 3.4 mmol/L, or a previous diagnosis of hypercholesterolemia or taking cholesterol-lowering medications [20].

### Blood DNA extraction and quality control

The blood samples were processed for cell lysis to release the genomic DNA. Blood cell DNA extraction was performed using the Qiagen DNeasy Blood and Tissue Kit, which was carried out in strict accordance with the standard protocol provided by the kit manufacturer. The concentration and purity of the purified DNA samples were determined using Qubit 3.0 Fluorometer and NanoDrop One spectrophotometer. To analyze DNA methylation, bisulfite conversion of the purified genomic DNA was performed. The bisulfite treatment converts unmethylated cytosines to uracils while preserving methylated cytosines. The conversion reaction was carried out according to the manufacturer's instructions. Throughout the extraction process, quality control measures were implemented, including monitoring sample handling, recording any deviations from the protocol, and assessing DNA quality through gel electrophoresis. To reduce variables in any type of experiment, we include both positive and negative controls in the experimental design. Positive controls are available as Internal Positive Control (IPC), which is spiked into samples before qPCR assay to confirm the absence of target sequence, inhibition, or a reaction cycling error. Negative controls are extracted at the same time as samples and are used to check the presence of possible contamination during the extraction steps.

### Blood DNA methylation analysis

Genome-wide DNA methylation profiles were obtained using the Illumina Infinium MethylationEPIC BeadChips following the manufacturer guide and protocol for Infinium MethylationEPIC array. Samples were randomized for each slide, plate, and the position on plate, based on covariates including age, sex, and BMI, to remove any potential bias on DNA methylation measurement from technically induced variation or confounding. Five hundred nanogram of genomic DNA from each blood sample was bisulfite converted using the EZ DNA Methylation Kit. DNA-BeadChip hybridization and single base extension were performed using a Freedom EVO robot. BeadChips were subsequently imaged using the iScan Microarray Scanner (Illumina), and Illumina.idat files were then processed with an R package named ChAMP [21]. Probes on chromosome X and Y and SNP-related probes were removed. The SNP list comes from a published paper [22]. Beta values were calculated corresponding to the ratio of the methylated signal over the sum signal, and *P* values were derived by comparing the sum signal to that of the background distribution. Betas with *P* values above than 0.01 were set to NA. Probes with less than 3 beads in at least 5% of samples per probe were filtered out. After quality control, beta values were normalized using a method named Beta Mixture Quantile (BMIQ) [23]. Batch effects were then corrected using an R package named ComBat [24]. The derived beta values were used for further analysis.

### Epigenome-wide association analysis and pathway analysis

A linear regression model was fitted to capture the correlation between genome-wide DNA methylation and the change of liver fat content, accomplished with the R package limma [25]. We conducted the association analysis to discover CpG positions significantly related to liver fat content (i.e., differentially methylated positions). We annotated the detected CpGs to protein-coding genes and CpG islands using an R package named IlluminaHumanMethylationEPICanno.ilm10b4.hg19 [26]. Then, we checked the enrichment of those genes in Kyoto Encyclopedia of Genes and Genomes (KEGG) pathways [27].

### Calculation of cell proportion in blood

Since heterogeneity in the composition of blood leukocyte cell types can confound the relationships between DNA methylation and phenotypes, we estimated the cell type abundance from methylation data using an R package named EpiDISH [28]. The percentages of seven different cell types (CD4 T cells, CD8 T cells, NK cells, B cells, monocytes, and neutrophil) were calculated by mapping the beta values of CpGs to the reference values according to the database provided by EpiDISH.

### Determination of DNA methylation age

DNA methylation age was calculated using the online age calculator (https://dnamage.genetics.ucla.edu/) developed by Horvath [11]. Normalized DNA methylation data were used as input for the algorithm, and additional normalization and imputation were performed by the age calculator for missing beta values. The list of predictive CpGs provided by Horvath was trained based on DNA methylation of 450 K Chips. In our case, the DNA methylation was obtained using MethylationEPIC BeadChips (i.e., 850 K); therefore, we only used the overlapped CpGs between 450 and 850 K as predictors.

### Statistical analysis

All statistical analyses were performed using R software version 3.6.2 and SPSS software version 15.0. The continuous parameters with normal distribution are presented as the means ± SD, and skewed parameters are presented as the median with the interquartile range (25–75%) given in parentheses. All skewed parameters were normalized using rank-based inverse normal transformation before analysis. The continuous data with normal distribution were compared using the Student’s *t*-tests or one-way analysis of covariance (ANOVA) and the categorical variables using the chi-square test. The quantitative correlations of DNA methylation age acceleration and its composite CpGs with the longitudinal changes of liver fat content were analyzed using Spearman correlation analysis. Multivariate linear and logistic regression analyses were used to investigate the associations of DNA methylation age acceleration with longitudinal change of liver fat content and the risk of NAFLD after adjustment for chronological age, sex, alcohol drinking, cigarette smoking, BMI, waist circumference, and different type blood cell counts at baseline. Components of metabolic syndrome, including FPG, SBP, TG, and HDL cholesterol, were further adjusted in the multivariate linear regression model relating the DNA methylation age acceleration to the change of liver fat content.

Multivariate logistic regression models including the conventional metabolic risk factors, key Horvath CpGs related to risk of NAFLD, and their combination were used to build scores for incident NAFLD. Variables entered the regression models were finally assessed by backward stepwise regression analysis to obtain the optimal NAFLD prediction scores. The receiver operating characteristic curve analyses were then performed to describe the diagnostic performance of the above NAFLD prediction scores for incidence of NAFLD after 4-year follow-up. Internal cross-validation was performed to validate the NAFLD prediction scores using a tenfold cross-validation technique that was repeated 200 times with R program. The areas under the ROC curve (AUROCs) were compared using the DeLong’s test. The low and high cutoff points were determined to predict incident NAFLD with sensitivity (probability that the score is positive for participants who will develop NAFLD) of at least 90% and with specificity (probability that the score is negative for participants who will not develop NAFLD) of at least 90% for all four prediction scores. All statistical analyses were two-sided, and *P* < 0.05 was considered statistically significant unless otherwise stated.

## Results

### Baseline characteristics

**Fig. 1:**
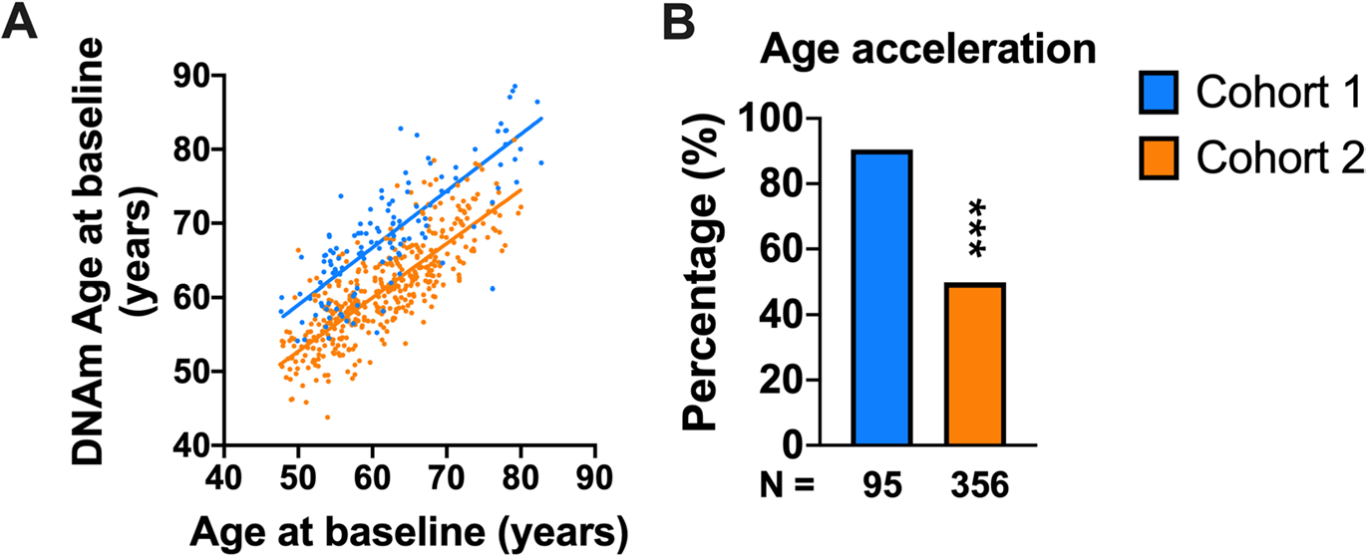
Chronological ages and DNA methylation ages in both cohorts. **A** Correlation between chronological ages and DNA methylation ages. **B** Proportion of participants with DNA methylation age acceleration in both cohorts

We analyzed two cohorts from a Chinese community-based population. Cohort 1 enrolled participants with normal plasma glucose at baseline but new-onset of type 2 diabetes at follow-up examination. Cohort 2 was a randomly sampled representative cohort from a community population. The chronological ages were highly correlated with the DNA methylation ages in both cohorts (*r* = 0.79, *P* < 0.001 and *r* = 0.81, *P* < 0.001, respectively), as shown in Fig. 1A. DNA methylation age was greater than chronological age in 86 (90.5%) participants from Cohort 1 and 163 (45.8%) participants from Cohort 2 (Fig. 1B). The participants from Cohort 1 had an average chronological age of 61.5 ± 8.4 years and an average DNA methylation age of 67.7 ± 8.0 years, and they had an age acceleration of 6.2 ± 5.1 years on average (*P* < 0.001) (Table 1). In comparison, the average chronological and DNA methylation age of participants from the randomly sampled Cohort 2 were 61.2 ± 7.3 years and 60.8 ± 6.3 years, respectively (Table 2). The prevalence of NAFLD, metabolic syndrome, hypertension, hypertriglyceridemia, and hypercholesterolemia was 31.6%, 28.9%, 30.5%, 18.9%, and 47.4% in Cohort 1 and 36.2%, 28.0%, 27.5%, 21.1%, and 47.4% in Cohort 2. All participants enrolled in Cohort 1 had no diabetes at baseline and developed type 2 diabetes during 4-year follow-up, while the baseline prevalence of type 2 diabetes in Cohort 2 was 18.0%.

**Table 1:** Metabolic characteristics of the participants divided by degrees of DNA methylation age acceleration from Cohort 1 (*n* = 95)

	Total	DNA methylation age acceleration			*P* value
		First tertile (< 4.0 years)	Second tertile (4.0–9.5 years)	Third tertile (≥ 9.5 years)	
No. of participants	95	33	32	30	
Male, *n* (%)	50 (52.6%)	12 (36.4%)	21 (65.6%)	17 (56.7%)	0.098
Cigarette smoking, *n* (%)	16 (16.8%)	4 (12.1%)	6 (18.8%)	6 (20.0%)	0.401
Alcohol drinking, *n* (%)	9 (9.5%)	1 (3.0%)	3 (9.4%)	5 (16.7%)	0.067
Chronological age, years	61.5 ± 8.4	63.4 ± 9.3	62.4 ± 8.8	58.6 ± 6.1	0.053
DNA methylation age, years	67.7 ± 8.0	64.4 ± 8.2	68.9 ± 8.5^a^	70.1 ± 6.1^a^	0.011
BMI, kg/m^2^	24.1 ± 2.4	24.1 ± 2.2	24.6 ± 2.5	23.7 ± 2.6^a^	0.349
Waist circumference, cm	84.0 ± 7.7	83.4 ± 7.5	86.0 ± 7.4^a^	82.6 ± 7.9	0.173
Systolic blood pressure, mmHg	134.1 ± 16.2	132.4 ± 13.0	134.6 ± 17.8	135.5 ± 18.0	0.738
Diastolic blood pressure, mmHg	78.2 ± 8.9	76.5 ± 9.5	79.1 ± 8.9	78.9 ± 8.4	0.433
Fasting plasma glucose, mmol/L	5.1 ± 0.5	5.0 ± 0.5	5.2 ± 0.5	5.0 ± 0.4	0.111
OGTT 2-h plasma glucose, mmol/L	6.2 ± 1.1	6.2 ± 1.1	6.0 ± 1.3	6.3 ± 1.1	0.654
HbA1c, %	5.6 ± 0.3	5.6 ± 0.3	5.6 ± 0.3	5.5 ± 0.3	0.464
Triglycerides, mmol/L	1.5 (1.1–2.0)	1.7 (1.1–2.0)	1.5 (1.2–1.9)	1.4 (1.1–2.2)	0.732
Cholesterol, mmol/L	5.2 ± 0.9	5.4 ± 0.7	4.9 ± 1.0	5.1 ± 0.9	0.070
HDL cholesterol, mmol/L	1.4 ± 0.3	1.4 ± 0.3	1.4 ± 0.4	1.3 ± 0.3	0.385
LDL cholesterol, mmol/L	3.0 ± 0.8	3.2 ± 0.6	2.8 ± 0.9	3.0 ± 0.8	0.075
ALT, U/L	16 (13–23)	15 (12–21)	19 (13–25)	16 (13–22)	0.118
AST, U/L	21 (18–24)	22 (20–25)	21 (19–23)	19 (17–22)	0.145
Liver fat content, %	4.7 (1.8–11.6)	3.1 (0.4–9.0)	4.9 (2.6–13.1)	7.2 (3.4–13.1)^a^	0.019

The continuous parameters with normal distribution were presented as the means ± SD, and skewed parameters were presented as the median with the interquartile range (25–75%) given in parentheses

*BMI* body mass index, *OGTT* oral glucose tolerance test, *ALT* alanine transaminase, *AST* aspartate transaminase, *NAFLD* non-alcoholic fatty liver disease

^a^*P* < 0.05 compared with participants with age acceleration < 4 years

**Table 2:** Baseline characteristics of the participants with and without DNA methylation age acceleration from Cohort 2 (*n* = 356)

	Total	Non-DNAm age acceleration	DNAm age acceleration	*P* value
No. of participants	356	193	163	
Male, *n* (%)	149 (41.9%)	77 (39.9%)	72 (44.2%)	0.415
Cigarette smoking, *n* (%)	57 (16.0%)	24 (12.4%)	33 (20.3%)	0.043
Alcohol drinking, *n* (%)	55 (15.4%)	17 (8.8%)	38 (23.3%)	< 0.001
Chronological age, years	61.2 ± 7.3	64.3 ± 6.8	57.5 ± 6.0	< 0.001
DNA methylation age, years	60.8 ± 6.3	60.6 ± 6.4	60.9 ± 6.3	0.666
BMI, kg/m^2^	23.9 ± 3.0	23.7 ± 3.0	24.1 ± 3.0	0.239
Height, cm	162.1 ± 7.9	161.5 ± 7.9	162.9 ± 7.8	0.078
Weight, kg	62.8 ± 9.8	61.8 ± 9.5	64.0 ± 10.0	0.038
Waist circumference, cm	83.1 ± 8.7	82.8 ± 8.3	83.3 ± 9.2	0.600
Systolic blood pressure, mmHg	130.7 ± 18.0	131.2 ± 17.8	130.0 ± 18.2	0.520
Diastolic blood pressure, mmHg	74.8 ± 10.3	73.4 ± 10.0	76.4 ± 10.5	0.007
Fasting plasma glucose, mmol/L	5.5 ± 1.3	5.5 ± 1.5	5.3 ± 1.1	0.169
OGTT 2-h plasma glucose, mmol/L	7.5 ± 2.9	7.6 ± 2.8	7.3 ± 3.1	0.263
HbA1c, %	5.8 ± 0.9	5.9 ± 1.1	5.7 ± 0.7	0.056
Triglycerides, mmol/L	1.4 (1.1–2.2)	1.6 ± 0.8	1.9 ± 1.3	0.009
Cholesterol, mmol/L	5.1 ± 0.9	5.1 ± 0.8	5.2 ± 0.9	0.089
HDL cholesterol, mmol/L	1.5 ± 0.4	1.5 ± 0.4	1.4 ± 0.4	0.213
LDL cholesterol, mmol/L	2.9 ± 0.8	2.8 ± 0.7	3.0 ± 0.8	0.097
ALT, U/L	16 (12–23)	16 (12–23)	16 (13–23)	0.723
AST, U/L	20 (18–24)	21 (18–25)	20 (17–23)	0.145
Liver fat content, %	5.7 (2.2–12.9)	5.6 (2.5–12.2)	6.5 (1.8–13.1)	0.814

The continuous parameters with normal distribution were presented as the means ± SD, and skewed parameters were presented as the median with the interquartile range (25–75%) given in parentheses

*DNAm* DNA methylation, *BMI* body mass index, *OGTT* oral glucose tolerance test, *ALT* alanine transaminase, *AST* aspartate transaminase

### DNA methylation age acceleration is associated with the risk of NAFLD

**Fig. 2:**
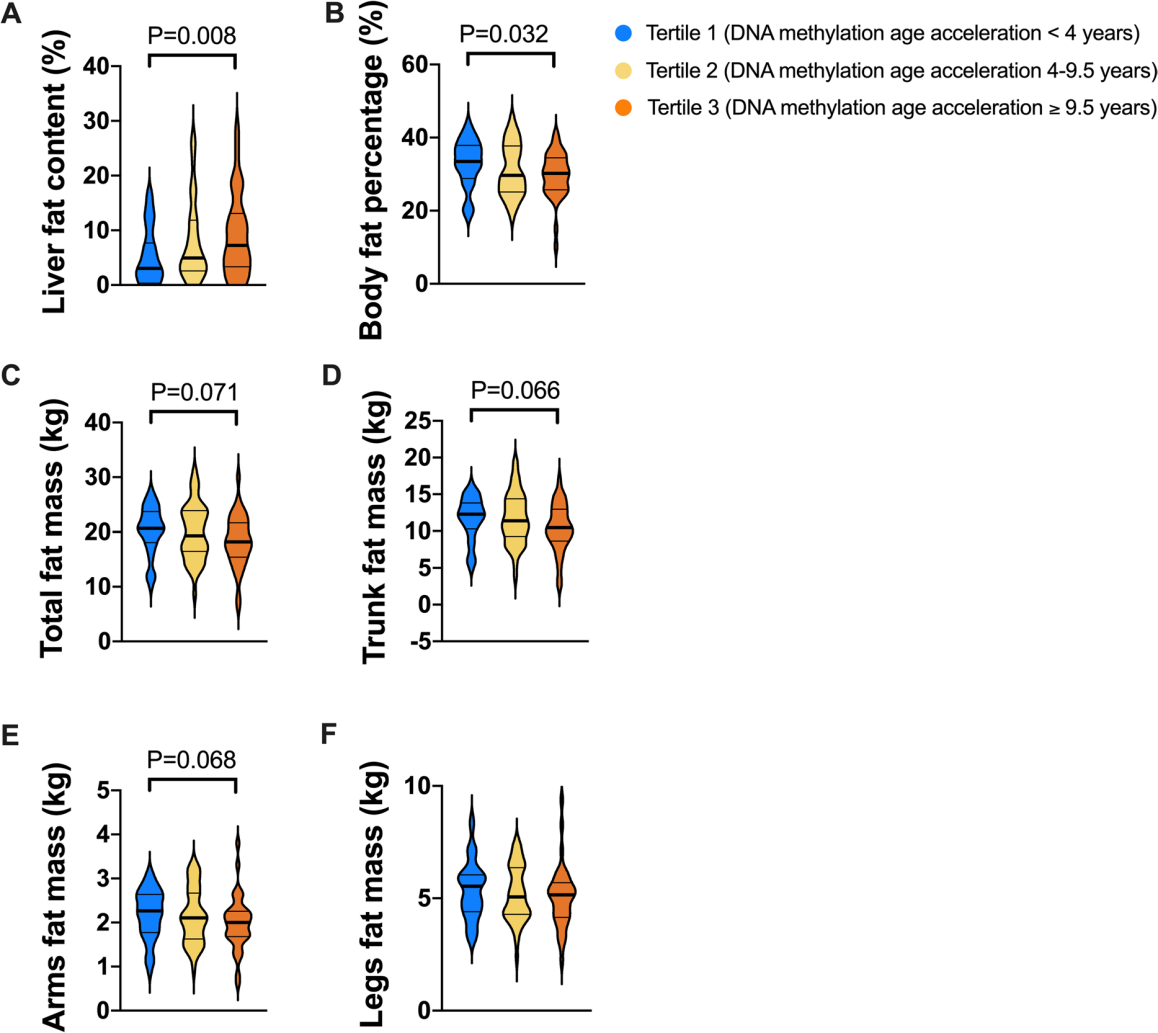
Relationship of DNA methylation age acceleration with liver fat content and whole-body and regional body fat masses. The comparison of **A** liver fat content, **B** body fat percentage, and **C**–**F** total, trunk, arms, and legs fat masses among the participants with tertiles of DNA methylation age acceleration

We divided the participants from Cohort 1 into three groups according to the tertiles of DNA methylation age acceleration: tertile 1, < 4.0 years; tertile 2, 4.0–9.5 years; and tertile 3, ≥ 9.5 years. As shown in Table 1, the baseline levels of BMI, waist circumference, blood pressure, plasma glucose, serum triglycerides, cholesterol, and liver enzymes had no significant difference among the participants with different degrees of age acceleration, except for a slightly lower BMI level in the third tertile and higher waist circumference in the second tertile group. Intriguingly, despite similar glucose and lipid metabolic parameters, the participants with highest tertile of DNA methylation age acceleration (≥ 9.5 years) had significantly higher liver fat content (7.2% vs 3.1%, *P* = 0.008) and lower body fat percentage (29.7% vs 33.0%, *P* = 0.032) than those with first tertile of age acceleration (< 4.0 years) (Fig. 2A, B). Moreover, the whole-body fat masses as well as fat masses at the trunk and arms sites also had a trend of reduction in participants with highest tertile of DNA methylation age acceleration (Fig. 2C–E). There was no difference in fat masses at the legs among the participants with different degrees of DNA methylation age acceleration (Fig. 2F). After gradual adjustment for chronological age, sex, alcohol drinking, cigarette smoking, BMI, waist circumference, and different type blood cell counts at baseline, participants with age acceleration ≥ 9.5 years still had significantly higher risk of NAFLD (OR, 4.55; 95% CI, 1.06–19.61) and MAFLD (OR, 5.57; 95% CI, 1.03–30.01) but not other metabolic diseases (metabolic syndrome, hypertension, hypertriglyceridemia, and hypercholesterolemia), as shown in Table 3.

**Table 3:** Association of DNA methylation age acceleration and risk of NAFLD and metabolic diseases in Cohort 1 (*n* = 95)

	DNAm age acceleration < 4.0 years (*N* = 33)	DNAm age acceleration 4.0–9.5 years (*N* = 32)	DNAm age acceleration ≥ 9.5 years (*N* = 30)	*P* for trend
NAFLD				
No. of cases, *n* (%)	7 (21.2%)	9 (28.1%)	13 (43.3%)	
BMI-adjusted OR (95% CI)	1 (reference)	1.22 (0.36–4.11)	3.92 (1.16–13.28)	0.029
Multivariate-adjusted OR (95% CI)	1 (reference)	1.07 (0.27–4.22)	4.55 (1.06–19.61)	0.042
MAFLD				
No. of cases, *n* (%)	6 (18.2%)	9 (28.1%)	11 (36.7%)	
BMI-adjusted OR (95% CI)	1 (reference)	1.42 (0.39–5.17)	3.50 (0.95–12.93)	0.060
Multivariate-adjusted OR (95% CI)	1 (reference)	1.55 (0.34–7.03)	5.57 (1.03–30.10)	0.045
Metabolic syndrome				
No. of cases, *n* (%)	11 (33.3%)	9 (28.1%)	5 (16.7%)	
BMI-adjusted OR (95% CI)	1 (reference)	0.59 (0.18–1.89)	0.39 (0.10–1.43)	0.146
Multivariate-adjusted OR (95% CI)	1 (reference)	0.68 (0.14–3.36)	0.72 (0.11–4.76)	0.672
Hypertension				
No. of cases, *n* (%)	10 (30.3%)	9 (28.1%)	10 (33.3%)	
BMI-adjusted OR (95% CI)	1 (reference)	0.81 (0.27–2.42)	1.25 (0.42–3.71)	0.708
Multivariate-adjusted OR (95% CI)	1 (reference)	0.92 (0.26–3.30)	1.25 (0.33–4.71)	0.741
Hypertriglyceridemia				
No. of cases, *n* (%)	7 (21.2%)	4 (12.5%)	7 (23.3%)	
BMI-adjusted OR (95% CI)	1 (reference)	0.45 (0.11–1.80)	1.23 (0.36–4.16)	0.779
Multivariate-adjusted OR (95% CI)	1 (reference)	0.27 (0.05–1.58)	1.10 (0.20–6.01)	0.791
Hypercholesterolemia				
No. of cases, *n* (%)	21 (63.6%)	10 (31.3%)	14 (46.7%)	
BMI-adjusted OR (95% CI)	1 (reference)	0.25 (0.09–0.72)	0.51 (0.18–1.39)	0.163
Multivariate-adjusted OR (95% CI)	1 (reference)	0.22 (0.06–0.75)	0.46 (0.13–1.55)	0.208

Chronological age, sex, alcohol drinking, cigarette smoking, BMI, waist circumference, and different type blood cell counts at baseline were adjusted in the multivariable regression model

*DNAm* DNA methylation

### DNA methylation age acceleration is correlated with longitudinal changes of LFC

**Fig. 3:**
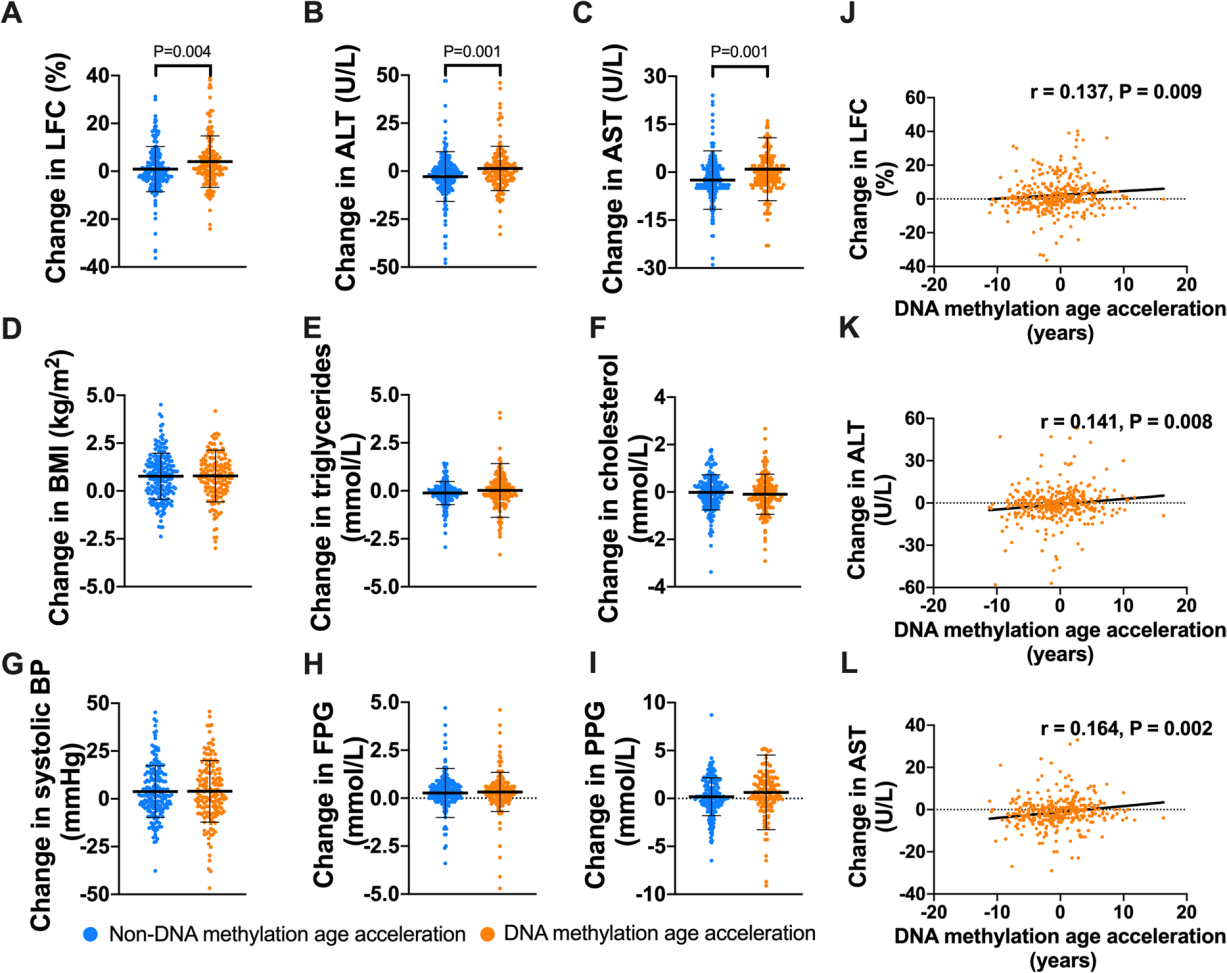
Relationship between DNA methylation age acceleration and longitudinal changes of liver fat content and related metabolic parameters. Comparison of the longitudinal changes in the levels of **A** liver fat content (LFC), **B** alanine transaminase (ALT), **C** aspartate transaminase (AST), **D** BMI, **E** triglycerides, **F** cholesterol, **G** systolic blood pressure, **H** fasting plasma glucose (FPG), and **I** post-load plasma glucose (PPG) between the participants with and without DNA methylation age acceleration. Linear correlations of DNA methylation age acceleration with changes in **J** LFC, **K** serum ALT, and **L** AST

The randomly sampled cohort (Cohort 2) consisted of 163 participants with DNA methylation age acceleration and 193 without DNA methylation age acceleration. Compared with the participants without DNA methylation age acceleration, participants with DNA methylation age acceleration had higher proportion of cigarette smoking (20.3% vs 12.4%, *P* = 0.043) and alcohol drinking (23.3% vs 8.8%, *P* < 0.001). As shown in Table 2, although the participants with DNA methylation age acceleration were younger in chronological age, their DNA methylation ages were similar to those without DNA methylation age acceleration at baseline. Most of metabolic parameters had no significant difference between the two groups, except for a higher body weight, DBP, and serum TG level in the age acceleration group. The LFC had no difference at baseline examination (5.6% vs 6.5%, *P* = 0.814). However, after a median of 4-year follow-up, the participants with age acceleration at baseline had significantly higher increase in LFC (4.0 ± 10.7 vs 0.9 ± 9.5%, *P* = 0.004), serum ALT (1.4 ± 11.5 vs − 2.9 ± 12.9U/L, *P* = 0.001), and AST (0.9 ± 9.9 vs − 2.5 ± 9.2U/L, *P* = 0.001) levels (Fig. 3A–C). In comparison, the change in BMI, serum TG and TC, SBP, FPG, and PPG had no difference between the participants with and without DNA methylation age acceleration (Fig. 3D–I). Quantitatively, DNA methylation age acceleration years at baseline were also positively correlated with longitudinal changes in LFC (*r* = 0.137, *P* = 0.009), ALT (*r* = 0.141, *P* = 0.008), and AST (*r* = 0.164, *P* = 0.002) after 4-year follow-up (Fig. 3J–L). Even after gradual adjustment for chronological age, sex, alcohol drinking, cigarette smoking, baseline BMI, waist circumference, FPG, SBP, TG, HDL-c, and different type blood cell counts, DNA methylation age acceleration still had a positive correlation with longitudinal changes in LFC (standardized β = 0.103, *P* = 0.041) and ALT (standardized β = 0.122, *P* = 0.007) and a trend of correlation with AST (standardized β = 0.085, *P* = 0.052) (Table 4).

**Table 4:** DNA methylation age acceleration and longitudinal changes in liver fat content and liver enzymes in Cohort 2 (*n* = 356)

DNA methylation age acceleration	Change in LFC			Change in ALT			Change in AST		
	Beta (Std)	Std β	*P* value	Beta (Std)	Std β	*P* value	Beta (Std)	Std β	*P* value
Model 1	0.24 (0.12)	0.137	0.009	0.39 (0.15)	0.141	0.008	0.32 (0.12)	0.164	0.002
Model 2	0.24 (0.12)	0.109	0.043	0.43 (0.16)	0.154	0.005	0.32 (0.12)	0.160	0.003
Model 3	0.27 (0.12)	0.117	0.025	0.39 (0.14)	0.145	0.001	0.20 (0.11)	0.105	0.019
Model 4	0.21 (0.11)	0.091	0.064	0.36 (0.14)	0.114	0.009	0.17 (0.11)	0.070	0.126
Model 5	0.24 (0.12)	0.103	0.041	0.34 (0.13)	0.122	0.007	0.18 (0.10)	0.085	0.052

Model 1: unadjusted

Model 2: adjusted for chronological age, sex, alcohol drinking, and cigarette smoking

Model 3: adjusted for covariates in Model 2 plus the baseline level of investigated parameter (LFC, ALT, or AST)

Model 4: adjusted for covariates in Model 3 plus BMI, waist circumference, FPG, SBP, TG, and HDL-c at baseline

Model 5: adjusted for covariates in Model 4 plus different type blood cell counts

### DNA methylation age acceleration may contribute to liver steatosis by mobilizing fat storage from the peripheral adipose tissue and accumulating them in the liver

**Fig. 4:**
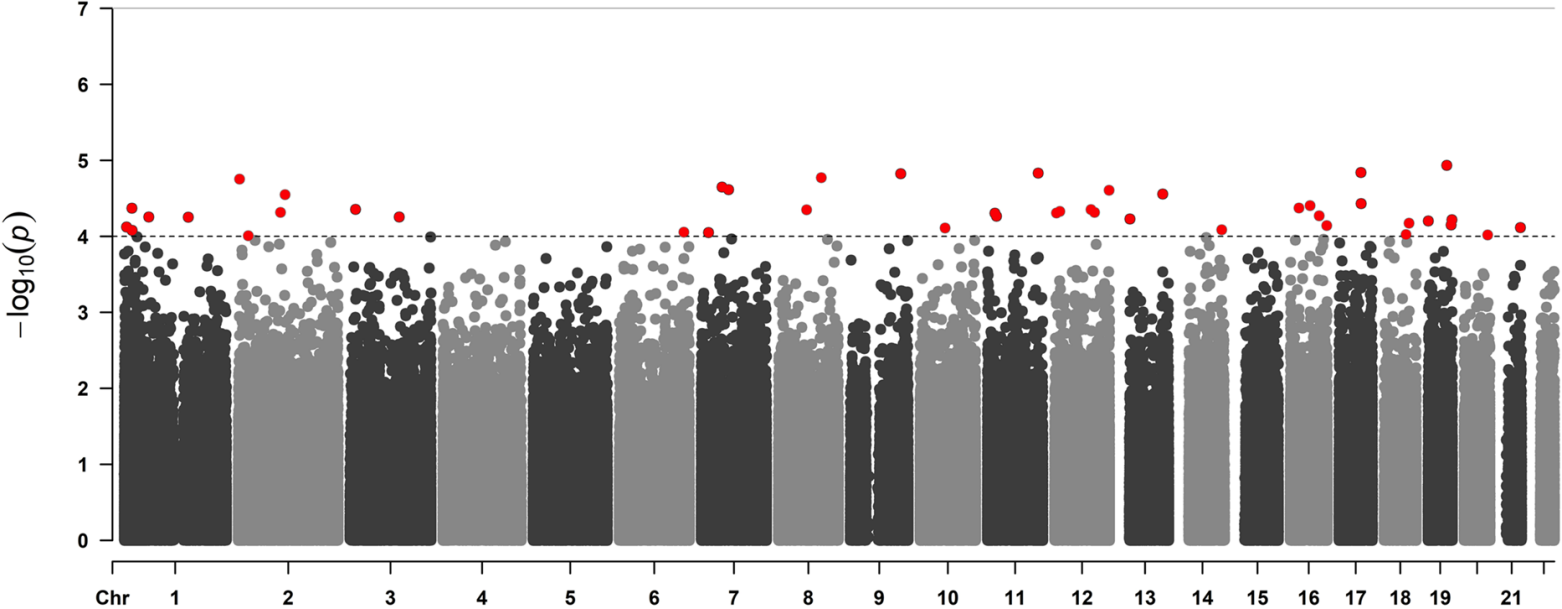
The Manhattan plot of the epigenome-wide association analysis. The CpGs that passed the threshold of *P* < 1 × 10^−4^ are marked red

We conducted epigenome-wide association analysis using all the DNA methylation data to globally detect differentially methylated positions. As a result, 44 related CpGs were identified at the *P* threshold of *P* < 1 × 10^−4^ (Fig. 4 and Supplementary table S1), although none of the CpGs was significant after Bonferroni correction. The enrichment analysis shows that the genes where the detected CpGs are located are enriched in wnt signaling pathway (KEGG: hsa04310; *P* = 8.76 × 10^−3^) and p53 signaling pathway (KEGG: hsa04115; *P* = 5.96 × 10^−4^), both of which have a close relationship with aging [29, 30], leading to a point that the correlation between DNA methylation and liver fat content is associated with aging.

**Fig. 5:**
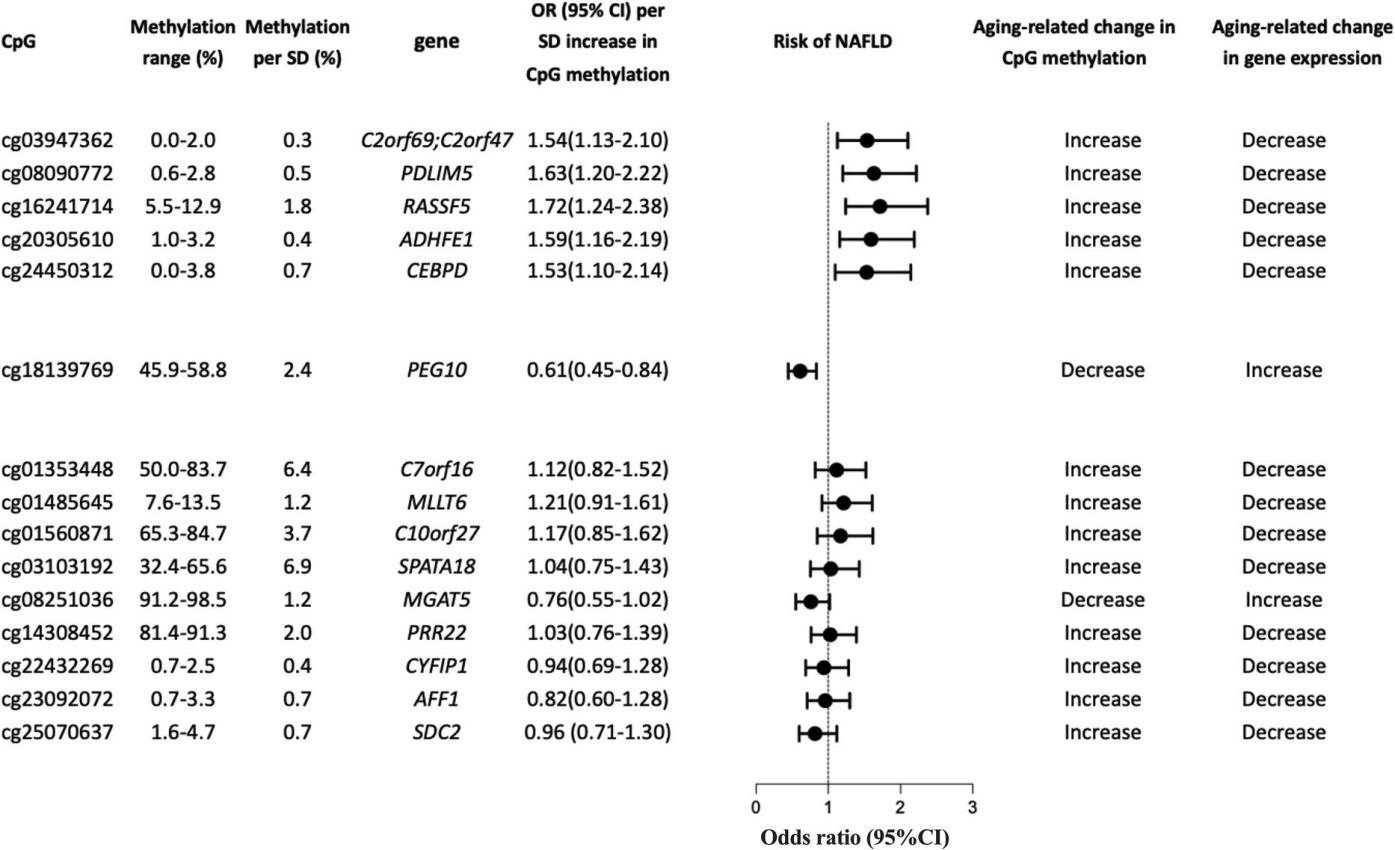
Six key Horvath CpGs of DNA methylation age predictors were associated with the risk of NAFLD. The odds ratio and 95% CI for risk of NAFLD per SD methylation level of each CpG site were estimated. The expression of genes regulated by the methylation of the CpG sites was also provided

Based on this understanding, we further focused on the exploration of associations between aging-related CpGs and liver fat content. We collected 353 CpGs defined as “aging clock” reported by Horvath et al. The associations between Horvath CpGs and the change of LFC were listed in Table S2. Among all Horvath CpGs sites, a total of 10 CpG (cg01353448, cg01485645, cg01560871, cg03103192, cg03947362, cg08090772, cg14038452, cg20305610, cg16241714, cg24450312) sites were positively associated with longitudinal changes in LFC, while 5 CpG (cg08251036, cg18139769, cg22432269, cg23092072, and cg25070637) sites were inversely associated with longitudinal changes in LFC. The genes regulated by the above 15 CpGs and their metabolic functions were listed in Table S3. Most of the significant sites were located in the promoter regions and CpG islands of their related genes (Figure S2), which indicated that the methylation of these CpG sites might down-regulate expression of their related functional genes. After adjustment for chronological age, sex, alcohol drinking, cigarette smoking, BMI, and different type blood cell counts at baseline, the association between the methylation levels of cg03947362, cg08090772, cg16241714, cg20305610, cg24450312, and cg18139769 and the risk of incident NAFLD remained significant (all *P* < 0.05), and a trend of inverse correlation was found between cg08251036 methylation and NAFLD (*P* = 0.055) (Fig. 5). As shown in Fig. 5 and Table S3, the cg03947362, cg08090772, cg16241714, cg20305610, and cg24450312 were strongly hypomethylated (0–12.9%) initially, and they were slightly methylated with the acceleration of DNA methylation age. Functionally, the increased methylation level of cg03947362 could disrupt the mitochondrial function and lead to liver glycogen and lipid storage through down-regulating the expression of *C2of69* gene [31], and the increased methylation of cg08090772 and cg16241714 inhibited the adipogenesis through down-regulating expression of *ADHFE1* and *CEBPD* genes [32, 33], thus leading to excessive release of fatty acids from adipose tissue. At the same time, the excessive fatty acids were further utilized for synthesis of triglycerides in the liver by up-regulating the expression of *PEG10* and *MGAT5* through aging-related demethylation in the moderately or highly methylated CpG sites, namely, cg18139769 and cg08251036 [34, 35]. Moreover, the methylation of cg20305610 and cg24450312 was also associated with increased risk of incident NAFLD, and their related *PDLIM5* and *RASSF5* genes have been reported to correlate with obesity, type 2 diabetes, and hypertension previously [36, 37]. Taken together, we found that several key Horvath CpGs defined as age predictors were associated with the risk of incident NAFLD, and their methylation levels could regulate the expression of genes that inhibited peripheral adipogenesis and lead to liver steatosis by mobilizing lipids from the adipose tissue and accumulating them in the liver, which was consistent with reduced body fat percentage and increased LFC observed in participants with highest tertile of DNA methylation age acceleration as shown in Fig. 2.

### Performance of DNA methylation age acceleration and related CpGs for prediction of NAFLD incidence

Prediction models for NAFLD were then established based on conventional clinical parameters (“NAFLD Clin Score”), CpGs related to LFC (“NAFLD Methylation Score”), and their combinations by applying backward multivariate regression analyses, as follows:

$$\mathrm{NAFLD\;Clin\;Score}=0.13\times \mathrm{BMI}+0.42\times \mathrm{TG}-0.42\times \mathrm{FPG}-1.26\times \mathrm{cigarette\;smoking}\left(\mathrm{yes}=1, \mathrm{no}=0\right)-0.99\times \mathrm{alcohol\;drinking}\left(\mathrm{yes}=1, \mathrm{no}=0\right)-2.49$$

$$\mathrm{NAFLD\;Methyl\;Score}=8.92\times \mathrm{cg}01560871+105.46\times \mathrm{cg}08090772+40.98\times \mathrm{cg}16241714-22.12\times \mathrm{cg}18139769+79.91\times \mathrm{cg}20305610-77.52\times \mathrm{cg}23092072-49.94\times \mathrm{cg}25070637+2.89$$

$$\mathrm{NAFLD\;ClinMethyl\;Score}=0.50\times \mathrm{TG}-0.48\times \mathrm{FPG}-2.35\times \mathrm{cigarette\;smoking}\left(\mathrm{yes}=1,\mathrm{no}=0\right)+11.14\times \mathrm{cg}01560871+128.21\times \mathrm{cg}08090772-27.16\times \mathrm{cg}18139769+87.87\times \mathrm{cg}20305610-98.31\times \mathrm{cg}23092072+4.76$$

**Fig. 6:**
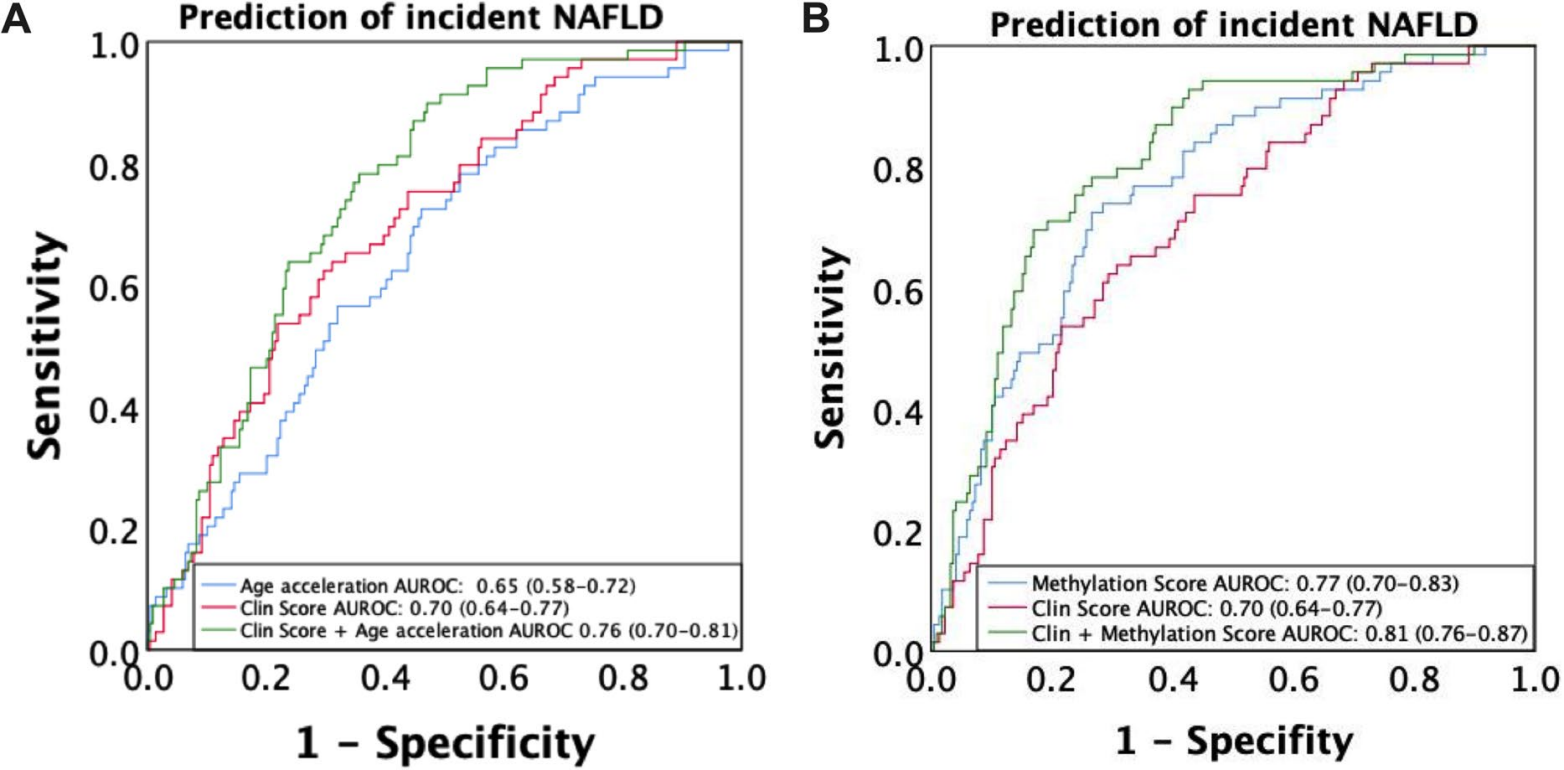
Diagnostic performance of the DNA methylation age acceleration and its key Horvath CpGs for prediction of NAFLD incidence. The receiver operating characteristic curve analyses were performed to describe the diagnostic performance of **A** the DNA methylation age acceleration and **B** the Horvath CpGs, conventional risk factors, and their combination for incidence of NAFLD

As shown in Fig. 6A, B, the DNA methylation age acceleration and the Horvath CpGs (“NAFLD Methyl Score”) could independently predict the incidence of NAFLD with AUROCs of 0.65 (0.58–0.72) and 0.77 (0.70–0.83), respectively. The AUROC of “NAFLD Clin Score” for prediction of NAFLD was 0.70 (0.64–0.77), and addition of DNA methylation age acceleration or related CpGs (“NAFLD ClinMethyl Score”) further improves the performance to predict NAFLD with AUROCs of 0.76 (0.70–0.81) (*P* = 0.039 vs “NAFLD Clin Score”) and 0.81 (0.76–0.87) (*P* < 0.001 vs “NAFLD Clin Score”), respectively. The cross-validation AUROCs of the “NAFLD Clin Score,” “NAFLD Clin Score” plus age acceleration, and “NAFLD ClinMethyl Score” were 0.69 (0.46–0.91), 0.74 (0.53–0.93), and 0.77 (0.58–0.96), respectively. Diagnostic performance of different NAFLD prediction models in terms of sensitivity and specificity and positive (PPV) and negative (NPV) predictive values under low and high cutoffs was listed in Table S4. The cutoffs for 90% sensitivity and 90% specificity was − 1.74 and − 0.63 for “NAFLD Clin Score” and − 1.60 and − 0.33 for “NAFLD ClinMethyl Score,” respectively. NPV was high (89.7–94.6%) in all four NAFLD prediction models, and “NAFLD ClinMethyl Score” could effectively exclude more participants with probability to develop NAFLD after 4-year follow-up than “NAFLD Clin Score” (48.1% vs 27.8%) by using the dual cutoff approach, leaving fewer participants in the gray zone between the two cutoffs (33.0% vs 56.4%).

## Discussion

NAFLD is a prevalent chronic disease and great health threat in the aging society. Our current study indicated that DNA methylation aging was an important risk factor for incident NAFLD in middle-aged and elderly population. Further analysis revealed that 6 CpGs of Horvath age predictors were associated with longitudinal changes in LFC, most of which were located on genes related to lipid metabolism that release fatty acids from peripheral adipose tissue by inhibiting adipogenesis and accumulate lipids in the liver by promoting hepatic lipogenesis and inhibiting lipid β-oxidation. Clinically, DNA methylation age acceleration and related Horvath CpGs could improve the accuracy to predict the incidence of NAFLD based on conventional risk factors.

DNA methylation age (“Horvath clock”) was first developed in 2013. Using 8000 samples from 82 Illumina DNA methylation array datasets, a predictor composed of 353 CpG sites was established to estimate methylation age of 51 tissues and cell types [11]. Previous studies indicated that DNA methylation age was independently associated with increased risk of metabolic syndrome [38], and this association seemed to be specific to worsening of lipid metabolism [39]. In our current study, we further found that DNA methylation age acceleration was cross-sectionally associated with higher risk of fatty liver disease. Even in the participants with similar baseline liver fat content, subjects with DNA methylation age acceleration also had higher risk of incident NAFLD than those without DNA methylation age acceleration after a median of 4-year follow-up, despite similar changes in BMI, blood pressure, plasma glucose and serum triglycerides, and cholesterol levels. Thus, hepatic lipid metabolism might be extremely vulnerable during the epigenetic aging process. Consistent with our findings, Horvath et al. found that adiposity increased DNA methylation age in a tissue-specific manner, and age acceleration played an extremely important role in liver-related comorbidities [12].

Our study indicated that DNA methylation data and clinical data were additive in the prediction of incident NAFLD. Usually, DNA methylation in the promoter regions and CpG islands down-regulates the expression of their related functional genes. Most of the Horvath CpGs of the NAFLD prediction score were located in the promoter regions and CpG islands and regulated the expression of genes in relation to liver and adipose lipid metabolism, which could not be completely explained by the clinical parameters.

However, it is also noticeable that no association was found between DNA methylation age acceleration in liver tissue and NAFLD Activity Score or liver steatosis grades in some studies previously [12], which seemed to be inconsistent with our results. In our current study, several key Horvath CpGs related to NAFLD (cg08090772, cg16241714, cg23092072, cg25070637) were functionally associated with peripheral adipogenesis and release of circulating fatty acids (Fig. 5 and Table S3). Therefore, our result supported that DNA methylation age acceleration promoted liver steatosis mainly through mechanisms relating to the interaction between the liver and peripheral adipose, and as such, peripheral blood DNA methylation could better reflect the metabolic status of peripheral adipose tissue and predict the risk of incident NAFLD, compared with the liver tissue DNA methylation. Consistent with our study, another study also reported that peripheral DNA methylation age acceleration could reflect the severity of liver fibrosis in patients with NASH [14].

In the current study, the participants with DNA methylation age acceleration were featured with an increase in liver fat content and a reduction in body fat percentage. The opposite changes in liver and peripheral adipose fat indicated an altered body fat distribution in the participants with DNA methylation age acceleration. It has been reported that with increasing age, fat distribution gradually shifts from subcutaneous to visceral areas [40], and the age-related reduction in capacity of subcutaneous fat to store lipids is a crucial mechanism leading to the NAFLD in the elderly population [41]. In the elderly people, the number of preadipocytes decreased remarkably [42], and the imbalance between lipolysis and lipogenesis in adipose tissue increased liver exposure to free fatty acids and lipotoxicity [43, 44]. Taken together, DNA methylation age acceleration promoted the development of NAFLD probably through altering the distribution between liver and peripheral adipose tissue.

The aged liver is characterized by an impairment of metabolic pathways involving hepatic lipid metabolism [45]. Senescent hepatocytes have increased lipid droplet accumulation [46], which was consistent with the increased NAFLD incidence observed in the elderly population. Compared with chronological age, the DNA methylation age (“Horvath age”) could better reflect the whole-body functional status metabolically. More importantly, the combination of DNA methylation age acceleration with conventional metabolic parameters could remarkably improve the performance to predict the risk of incident NAFLD. However, regarding the cost and reproducibility of the DNA methylation examination, it is important to establish whether the use of NAFLD ClinMethyl Score is cost-effective. Therefore, the current study is still preliminary to show the potential to use DNA methylation age for prediction of NAFLD.

Limitations of the current study should be considered when interpreting the results. First, the histological information for liver steatosis, inflammation, and fibrosis was not available, and the LFC was quantified using a quantitative ultrasound method that was not as accurate as liver biopsy or proton magnetic resonance spectroscopy (1H-MRS) [17]. Second, the NAFLD prediction score was derived and internally validated using tenfold cross-validation in Chinese middle-aged and elderly adults; further studies were still required to expand the conclusion to participants from different ethnicities and age groups.

## Conclusion

DNA methylation age acceleration was a novel epigenetic risk factor for NAFLD and could predict the incidence of NAFLD independent of all conventional metabolic risk factors. Functionally, NAFLD-related CpGs of DNA methylation age predictors were likely to regulate the release of fatty acids from peripheral adipose tissue by inhibiting adipogenesis and the accumulation of lipids in the liver by promoting hepatic lipogenesis and inhibiting lipid β-oxidation. Combination of DNA methylation age acceleration and related Horvath CpGs with conventional metabolic risk factors could improve the accuracy in predicting the incidence of NAFLD in middle-aged and elderly population.

### Supplementary Information

Below is the link to the electronic supplementary material.

Supplementary file1 (DOCX 385 KB)

## Data Availability

The data generated and analysed during the current study are not publicly available due to the relevant policy of data management from the sponsors of Chinese national and local government, but are available from the corresponding authors upon reasonable request and with permission of Chinese national and local government.

## Abbreviations

ALT: Alanine transaminase
ANOVA: One-way analysis of covariance
AST: Aspartate transaminase
AUROC: Area under the ROC curve
BMI: Body mass index
DBP: Diastolic blood pressure
FPG: Fasting plasma glucose
HDL: High-density lipoprotein
LDL: Low-density lipoprotein
LFC: Liver fat content
NAFLD: Non-alcoholic fatty liver disease
NASH: Non-alcoholic steatohepatitis
NPV: Negative predictive value
MAFLD: Metabolic associated fatty liver disease
OGTT: Oral glucose tolerance test
PPG: Post-load plasma glucose
PPV: Positive predictive value
ROI: Region of interest
SBP: Systolic blood pressure
TC: Total cholesterol
TG: Triglycerides
