# Supplementary material for: DNA methylation age acceleration contributes to the development and prediction of non-alcoholic fatty liver disease
Source: GeroScience. 2023 Aug 22;46(4):3525–42. doi: 10.1007/s11357-023-00903-5 (PMC11226581; doi:10.1007/s11357-023-00903-5)
Supplement: Supplementary file 1 — Supplementary file1 (DOCX 385 KB) [file 11357_2023_903_MOESM1_ESM.docx]

DNA methylation age acceleration contributes to the development and prediction of non-alcoholic fatty liver disease

(Supplementary Materials)

Mingfeng Xia^1*^, Wenran Li^2*^, Huandong Lin^1^, Hailuan Zeng^1^, Shuai Ma^1^, Qi Wu^1^, Hui Ma^4^, Xiaoming Li^1^, Baishen Pan^5^, Jian Gao^6^, Yu Hu^4^, Yun Liu^7^, Sijia Wang^2,3$^, Xin Gao^1$^

^1^Department of Endocrinology and Metabolism, Zhongshan Hospital and Fudan Institute for Metabolic Diseases, Human Phenome Institute, Fudan University, Shanghai 201203, China

^2^CAS Key Laboratory of Computational Biology, Shanghai Institute of Nutrition and Health, University of Chinese Academy of Sciences, Chinese Academy of Sciences, Shanghai 200031, China

^3^Center for Excellence in Animal Evolution and Genetics, Chinese Academy of Sciences, Kunming 650223, China.

^4^Department of Geriatrics, Zhongshan Hospital, Fudan University, Shanghai 200032, China

^5^Department of Laboratory Medicine, Zhongshan Hospital, Fudan University, Shanghai 200032, China

^6^Department of Nutrition, Zhongshan Hospital of Fudan University, Shanghai 200032, China

^7^Department of Biochemistry and Molecular Biology, School of Basic Medical Sciences and Zhongshan Hospital, Fudan University, Shanghai, China.

^*^These authors contributed equally to this work.

Table of contents

Table S1…………………………………………………...………………………………....…2

Table S2…………………………………………..………………………………………….....4

Table S3…………………………………………..…………………………………………....12

Table S4…………………………………………..…………………………………………....15

Figure S1………………………………………..………………………………………….......16

Figure S2………………………………………..………………………………………….......17

Table S1 Annotation of CpGs significantly associated with longitudinal change of liver fat content at the threshold of *P*<1×10^-4^.

| **CpG** | **Chr** | **Pos** | **Relation to Island** | **UCSC RefGene Name** | **UCSC RefGene Group** | **Effect Size** | ***P* Value** |
| --- | --- | --- | --- | --- | --- | --- | --- |
| cg22330935 | chr19 | 45586368 | Shelf | GEMIN7 | 5'UTR | -0.02894 | 1.16E-05 |
| cg25826457 | chr17 | 53342602 | Island | HLF | 5'UTR;1stExon | -0.02204 | 1.45E-05 |
| cg17003376 | chr11 | 123613212 | Shore | ZNF202 | TSS1500 | 0.026851 | 1.48E-05 |
| cg27442871 | chr9 | 122984045 | OpenSea |  |  | -0.03178 | 1.50E-05 |
| cg21593967 | chr8 | 104427890 | Shore | SLC25A32;DCAF13 | TSS1500;Body | 0.025548 | 1.69E-05 |
| cg14446504 | chr2 | 3291358 | Island | TSSC1 | Body | -0.02305 | 1.77E-05 |
| cg24792317 | chr7 | 50340637 | Shelf |  |  | -0.03194 | 2.25E-05 |
| cg11744031 | chr7 | 66423657 | OpenSea |  |  | 0.031475 | 2.43E-05 |
| cg17072811 | chr12 | 132606794 | Shelf | EP400NL | Body | 0.026492 | 2.48E-05 |
| cg16033633 | chr13 | 100624855 | Shore | ZIC5 | TSS1500 | -0.03105 | 2.78E-05 |
| cg04293501 | chr2 | 114037465 | Shore | PAX8 | TSS1500 | 0.026975 | 2.83E-05 |
| cg27617435 | chr17 | 53628652 | OpenSea |  |  | 0.029743 | 3.71E-05 |
| cg05311589 | chr16 | 48399731 | Island | SIAH1 | 5'UTR;1stExon | -0.03065 | 3.94E-05 |
| cg15875735 | chr16 | 21162515 | OpenSea | DNAH3 | Body | 0.026285 | 4.23E-05 |
| cg08655330 | chr1 | 19132135 | OpenSea |  |  | 0.028021 | 4.27E-05 |
| cg00498816 | chr3 | 13665022 | OpenSea | FBLN2 | Body | -0.02873 | 4.42E-05 |
| cg21131402 | chr12 | 88423891 | OpenSea | C12orf50 | TSS1500 | -0.02429 | 4.44E-05 |
| cg25153363 | chr8 | 68658656 | OpenSea | CPA6 | TSS200 | -0.02799 | 4.48E-05 |
| cg01489508 | chr12 | 12377151 | OpenSea | LRP6 | Body | -0.03059 | 4.70E-05 |
| cg05695631 | chr12 | 97305532 | OpenSea | NEDD1 | 5'UTR;Body | -0.03055 | 4.83E-05 |
| cg09342606 | chr2 | 102802603 | Shore | IL1RL2 | TSS1500 | -0.03073 | 4.85E-05 |
| cg13404421 | chr12 | 4382181 | Island | CCND2 | TSS1500 | -0.02954 | 4.94E-05 |
| cg16010467 | chr11 | 18195086 | OpenSea | MRGPRX4 | 1stExon | -0.02659 | 4.95E-05 |
| cg08238283 | chr16 | 70322942 | Shore | DDX19B;AARS | TSS1500;5'UTR | -0.02432 | 5.37E-05 |
| cg07083101 | chr11 | 22067714 | OpenSea |  |  | 0.030395 | 5.45E-05 |
| cg12067687 | chr1 | 60280868 | Island | HOOK1 | Body | -0.03083 | 5.55E-05 |
| cg07352158 | chr3 | 120170646 | Shore | FSTL1 | TSS1500 | 0.028318 | 5.56E-05 |
| cg22377142 | chr1 | 156265275 | Shelf | C1orf85 | Body | -0.02817 | 5.61E-05 |
| cg21794940 | chr13 | 20650664 | OpenSea | ZMYM2 | Body | -0.03007 | 5.89E-05 |
| cg13752138 | chr19 | 58264873 | OpenSea | ZNF776 | Body | -0.02776 | 6.05E-05 |
| cg26820693 | chr19 | 306492 | Island | MIER2 | 3'UTR | 0.024843 | 6.26E-05 |
| cg22720686 | chr18 | 60506128 | OpenSea | PHLPP1 | ExonBnd;Body | -0.03109 | 6.71E-05 |
| cg01692075 | chr19 | 56164060 | Island | CCDC106;U2AF2 | Body;TSS1500 | 0.027739 | 7.09E-05 |
| cg16596367 | chr16 | 89070757 | Island |  |  | -0.03026 | 7.21E-05 |
| cg09159106 | chr1 | 6007727 | OpenSea | NPHP4 | Body;5'UTR | 0.028934 | 7.52E-05 |
| cg08559978 | chr21 | 45302265 | OpenSea | AGPAT3 | 5'UTR | -0.02827 | 7.65E-05 |
| cg07062043 | chr10 | 60935910 | Island | PHYHIPL | TSS1500 | -0.02383 | 7.77E-05 |
| cg11732230 | chr14 | 100434776 | Shelf |  |  | 0.024025 | 8.21E-05 |
| cg23759600 | chr1 | 19334771 | OpenSea |  |  | 0.027038 | 8.33E-05 |
| cg14783044 | chr6 | 157423398 | OpenSea | ARID1B | Body | -0.02461 | 8.80E-05 |
| cg07098986 | chr7 | 17497154 | OpenSea | KCCAT333 | Body | 0.025179 | 8.93E-05 |
| cg11549065 | chr18 | 53156635 | OpenSea | TCF4 | Body | -0.0241 | 9.47E-05 |
| cg01153174 | chr20 | 57154319 | OpenSea | APCDD1L-AS1 | Body | 0.023842 | 9.59E-05 |
| cg07979316 | chr2 | 24306908 | Shore | TP53I3 | Body | -0.02814 | 9.79E-05 |

| Table S2 Correlations of composite CpG of Horvath clock with longitudinal change of liver fat content. | | |
| --- | --- | --- |
|  | **Longitudinal change of liver fat content** | |
|  | **Correlation coefficient** | **P value** |
| **cg22432269** | **-0.200** | **0.003** |
| **cg14308452** | **0.168** | **0.013** |
| **cg01485645** | **0.165** | **0.014** |
| **cg23092072** | **-0.161** | **0.017** |
| **cg20305610** | **0.154** | **0.022** |
| **cg16241714** | **0.152** | **0.024** |
| **cg25070637** | **-0.151** | **0.024** |
| **cg08251036** | **-0.146** | **0.030** |
| **cg01353448** | **0.145** | **0.032** |
| **cg24450312** | **0.145** | **0.032** |
| **cg03103192** | **0.142** | **0.034** |
| **cg01560871** | **0.138** | **0.041** |
| **cg18139769** | **-0.137** | **0.042** |
| **cg08090772** | **0.135** | **0.046** |
| **cg03947362** | **0.134** | **0.047** |
| cg02489552 | 0.131 | 0.052 |
| cg14723032 | 0.129 | 0.055 |
| cg18055007 | 0.127 | 0.058 |
| cg22613010 | -0.127 | 0.060 |
| cg13975369 | -0.126 | 0.062 |
| cg16358826 | 0.122 | 0.070 |
| cg06738602 | 0.120 | 0.076 |
| cg15661409 | -0.120 | 0.076 |
| cg12946225 | 0.117 | 0.083 |
| cg13836627 | 0.113 | 0.092 |
| cg17274064 | 0.113 | 0.092 |
| cg13269407 | 0.113 | 0.095 |
| cg02332492 | 0.109 | 0.106 |
| cg26394940 | 0.108 | 0.108 |
| cg15341340 | -0.107 | 0.112 |
| cg03167275 | -0.107 | 0.113 |
| cg22568540 | 0.105 | 0.121 |
| cg05921699 | 0.104 | 0.123 |
| cg15381769 | 0.104 | 0.124 |
| cg07663789 | -0.103 | 0.126 |
| cg01570885 | -0.101 | 0.135 |
| cg26620959 | 0.101 | 0.136 |
| cg10281002 | 0.099 | 0.142 |
| cg17285325 | 0.097 | 0.151 |
| cg10377274 | 0.096 | 0.156 |
| cg13931228 | 0.095 | 0.159 |
| cg21801378 | 0.094 | 0.162 |
| cg26297688 | 0.094 | 0.163 |
| cg13899108 | -0.092 | 0.174 |
| cg12616277 | 0.091 | 0.178 |
| cg15988232 | 0.089 | 0.186 |
| cg12373771 | 0.088 | 0.192 |
| cg14060828 | 0.088 | 0.192 |
| cg24254120 | 0.086 | 0.200 |
| cg12941369 | -0.086 | 0.201 |
| cg25657834 | 0.086 | 0.203 |
| cg26842024 | 0.085 | 0.207 |
| cg03682823 | -0.084 | 0.212 |
| cg20914508 | -0.084 | 0.214 |
| cg22736354 | 0.084 | 0.214 |
| cg20999813 | -0.083 | 0.218 |
| cg07770222 | 0.083 | 0.222 |
| cg25411725 | 0.082 | 0.222 |
| cg06493994 | -0.082 | 0.223 |
| cg21370143 | 0.082 | 0.225 |
| cg07408456 | 0.082 | 0.226 |
| cg16168311 | -0.081 | 0.228 |
| cg03565323 | -0.081 | 0.231 |
| cg04528819 | 0.081 | 0.231 |
| cg26453588 | 0.080 | 0.234 |
| cg18984151 | 0.080 | 0.236 |
| cg02275294 | 0.079 | 0.240 |
| cg14258236 | 0.079 | 0.240 |
| cg06144905 | 0.079 | 0.241 |
| cg22809047 | 0.078 | 0.248 |
| cg03286783 | -0.077 | 0.256 |
| cg05442902 | -0.076 | 0.259 |
| cg08030082 | 0.076 | 0.259 |
| cg04452713 | -0.076 | 0.260 |
| cg23180365 | -0.076 | 0.260 |
| cg02479575 | -0.076 | 0.261 |
| cg26372517 | 0.075 | 0.268 |
| cg01968178 | 0.075 | 0.270 |
| cg09418283 | -0.074 | 0.273 |
| cg07337598 | 0.074 | 0.275 |
| cg15185286 | -0.073 | 0.280 |
| cg17686885 | 0.072 | 0.285 |
| cg01644850 | -0.072 | 0.288 |
| cg16034652 | 0.071 | 0.294 |
| cg15804973 | 0.070 | 0.300 |
| cg26045434 | 0.069 | 0.308 |
| cg16579101 | -0.069 | 0.310 |
| cg18956095 | 0.069 | 0.311 |
| cg25683012 | -0.068 | 0.316 |
| cg09509673 | 0.068 | 0.317 |
| cg08124722 | 0.068 | 0.318 |
| cg26003813 | 0.067 | 0.318 |
| cg17589341 | 0.067 | 0.320 |
| cg26043391 | 0.066 | 0.328 |
| cg18983672 | 0.066 | 0.330 |
| cg09722555 | 0.065 | 0.333 |
| cg02071305 | 0.065 | 0.337 |
| cg25166896 | -0.063 | 0.350 |
| cg20761322 | 0.063 | 0.354 |
| cg23941599 | -0.062 | 0.358 |
| cg03588357 | 0.062 | 0.360 |
| cg05675373 | 0.062 | 0.363 |
| cg19722847 | -0.061 | 0.364 |
| cg08771731 | -0.061 | 0.365 |
| cg03270204 | 0.061 | 0.369 |
| cg04836038 | 0.061 | 0.369 |
| cg01262913 | -0.061 | 0.371 |
| cg25505610 | 0.060 | 0.372 |
| cg12830694 | 0.060 | 0.374 |
| cg05755779 | 0.060 | 0.375 |
| cg26824091 | 0.060 | 0.375 |
| cg10523019 | 0.060 | 0.378 |
| cg25771195 | 0.059 | 0.381 |
| cg25552492 | -0.059 | 0.383 |
| cg10266490 | 0.059 | 0.384 |
| cg02335441 | 0.059 | 0.385 |
| cg16419345 | -0.058 | 0.392 |
| cg15547534 | 0.058 | 0.393 |
| cg05847778 | 0.056 | 0.406 |
| cg10486998 | 0.056 | 0.408 |
| cg20692569 | -0.056 | 0.409 |
| cg04126866 | -0.055 | 0.415 |
| cg26005082 | -0.055 | 0.419 |
| cg24888049 | 0.054 | 0.421 |
| cg21395782 | -0.054 | 0.424 |
| cg13129046 | 0.053 | 0.429 |
| cg14597908 | -0.053 | 0.430 |
| cg23124451 | 0.053 | 0.432 |
| cg08413469 | -0.053 | 0.437 |
| cg01656216 | 0.052 | 0.439 |
| cg14992253 | 0.052 | 0.439 |
| cg15974053 | -0.052 | 0.443 |
| cg04121983 | -0.051 | 0.450 |
| cg14727952 | 0.051 | 0.451 |
| cg21211748 | -0.051 | 0.452 |
| cg00431549 | 0.051 | 0.454 |
| cg23786576 | -0.051 | 0.454 |
| cg23517605 | 0.050 | 0.457 |
| cg25101936 | -0.050 | 0.458 |
| cg12985418 | -0.050 | 0.460 |
| cg26723847 | 0.050 | 0.461 |
| cg12768605 | 0.050 | 0.462 |
| cg06952310 | 0.050 | 0.464 |
| cg14408969 | -0.049 | 0.466 |
| cg16984944 | -0.049 | 0.466 |
| cg19346193 | 0.049 | 0.466 |
| cg05960024 | 0.049 | 0.472 |
| cg14424579 | -0.049 | 0.472 |
| cg07158339 | 0.048 | 0.475 |
| cg16547529 | -0.047 | 0.488 |
| cg24262469 | 0.046 | 0.495 |
| cg20828084 | -0.046 | 0.496 |
| cg18328933 | 0.046 | 0.498 |
| cg03891319 | -0.045 | 0.502 |
| cg18180783 | -0.045 | 0.504 |
| cg19420968 | -0.045 | 0.504 |
| cg27015931 | -0.045 | 0.506 |
| cg04094160 | 0.045 | 0.508 |
| cg04474832 | 0.045 | 0.508 |
| cg25928579 | 0.045 | 0.508 |
| cg20240860 | 0.045 | 0.509 |
| cg01459453 | 0.044 | 0.511 |
| cg19305227 | 0.044 | 0.511 |
| cg25159610 | -0.044 | 0.511 |
| cg17960516 | 0.044 | 0.512 |
| cg08965235 | -0.044 | 0.513 |
| cg12351433 | 0.044 | 0.516 |
| cg04268405 | 0.044 | 0.518 |
| cg09722397 | 0.044 | 0.519 |
| cg11025793 | 0.044 | 0.520 |
| cg06361108 | -0.043 | 0.525 |
| cg25564800 | -0.042 | 0.531 |
| cg26456957 | 0.042 | 0.538 |
| cg14654875 | -0.041 | 0.547 |
| cg03330058 | 0.041 | 0.549 |
| cg04084157 | 0.041 | 0.549 |
| cg08186124 | 0.040 | 0.555 |
| cg11314684 | 0.040 | 0.558 |
| cg05365729 | -0.039 | 0.566 |
| cg09441152 | -0.039 | 0.566 |
| cg19692710 | 0.039 | 0.568 |
| cg00374717 | 0.038 | 0.569 |
| cg23662675 | -0.038 | 0.569 |
| cg09885951 | -0.038 | 0.570 |
| cg05250458 | -0.038 | 0.574 |
| cg24081819 | 0.038 | 0.576 |
| cg13038560 | 0.038 | 0.577 |
| cg02364642 | 0.037 | 0.580 |
| cg06557358 | 0.037 | 0.586 |
| cg09646392 | -0.036 | 0.597 |
| cg19044674 | 0.036 | 0.599 |
| cg06836772 | 0.035 | 0.604 |
| cg00168942 | 0.035 | 0.607 |
| cg10940099 | -0.035 | 0.608 |
| cg27092035 | -0.034 | 0.618 |
| cg06513075 | 0.033 | 0.623 |
| cg07388493 | 0.033 | 0.627 |
| cg09133026 | 0.033 | 0.628 |
| cg22679120 | -0.032 | 0.631 |
| cg01511567 | -0.032 | 0.632 |
| cg09191327 | -0.032 | 0.635 |
| cg17063929 | 0.032 | 0.639 |
| cg27494383 | 0.031 | 0.643 |
| cg03019000 | -0.031 | 0.649 |
| cg01027805 | 0.031 | 0.650 |
| cg11299964 | 0.029 | 0.665 |
| cg16744741 | -0.029 | 0.669 |
| cg25148589 | -0.029 | 0.671 |
| cg22171829 | -0.029 | 0.672 |
| cg14175438 | -0.028 | 0.683 |
| cg08434234 | -0.027 | 0.686 |
| cg06688848 | 0.027 | 0.687 |
| cg21378206 | -0.027 | 0.691 |
| cg09019938 | -0.026 | 0.699 |
| cg20524216 | 0.026 | 0.706 |
| cg21460081 | 0.025 | 0.714 |
| cg10045881 | 0.025 | 0.715 |
| cg22901840 | 0.025 | 0.716 |
| cg24126851 | 0.024 | 0.718 |
| cg07498421 | 0.024 | 0.728 |
| cg27377450 | -0.024 | 0.728 |
| cg06121469 | 0.023 | 0.730 |
| cg01027739 | -0.023 | 0.731 |
| cg16150435 | 0.023 | 0.735 |
| cg01820374 | -0.023 | 0.739 |
| cg26845300 | 0.022 | 0.742 |
| cg02085507 | 0.022 | 0.748 |
| cg24580001 | -0.022 | 0.750 |
| cg26162695 | -0.021 | 0.760 |
| cg22006386 | 0.020 | 0.766 |
| cg22289837 | 0.020 | 0.768 |
| cg09118625 | -0.020 | 0.769 |
| cg17338403 | -0.020 | 0.770 |
| cg22449114 | -0.020 | 0.771 |
| cg21870884 | 0.019 | 0.773 |
| cg09809672 | -0.019 | 0.781 |
| cg18031008 | 0.018 | 0.786 |
| cg15262928 | 0.018 | 0.787 |
| cg20947775 | 0.018 | 0.787 |
| cg07849904 | -0.018 | 0.791 |
| cg13828047 | -0.017 | 0.801 |
| cg02580606 | -0.017 | 0.807 |
| cg19008809 | -0.017 | 0.807 |
| cg02047577 | 0.016 | 0.809 |
| cg06926735 | -0.016 | 0.810 |
| cg14423778 | 0.016 | 0.812 |
| cg13854874 | -0.016 | 0.817 |
| cg24116886 | 0.015 | 0.820 |
| cg05903609 | -0.015 | 0.825 |
| cg01407797 | 0.015 | 0.827 |
| cg12413566 | 0.014 | 0.834 |
| cg02154074 | 0.014 | 0.837 |
| cg19724470 | -0.014 | 0.839 |
| cg17853587 | 0.014 | 0.840 |
| cg14163776 | 0.014 | 0.841 |
| cg13319175 | -0.013 | 0.842 |
| cg22190114 | -0.013 | 0.846 |
| cg27202708 | 0.013 | 0.848 |
| cg22637507 | 0.013 | 0.852 |
| cg25781123 | 0.012 | 0.854 |
| cg13547237 | 0.012 | 0.855 |
| cg17655614 | 0.012 | 0.856 |
| cg27169020 | -0.012 | 0.865 |
| cg11388238 | -0.011 | 0.868 |
| cg14894144 | 0.011 | 0.871 |
| cg14501253 | -0.011 | 0.873 |
| cg00091693 | 0.011 | 0.874 |
| cg13302154 | -0.011 | 0.874 |
| cg27544190 | 0.011 | 0.874 |
| cg00075967 | 0.010 | 0.877 |
| cg14658362 | 0.010 | 0.878 |
| cg07291563 | -0.010 | 0.883 |
| cg25809905 | 0.010 | 0.885 |
| cg24834740 | 0.010 | 0.886 |
| cg24899750 | -0.010 | 0.887 |
| cg10345936 | 0.010 | 0.888 |
| cg07285276 | 0.009 | 0.890 |
| cg16899442 | -0.009 | 0.890 |
| cg19853760 | 0.009 | 0.895 |
| cg21305265 | 0.009 | 0.899 |
| cg19478743 | -0.009 | 0.900 |
| cg02827112 | -0.008 | 0.908 |
| cg17099569 | 0.008 | 0.908 |
| cg18440048 | 0.008 | 0.908 |
| cg00436603 | -0.008 | 0.911 |
| cg03578041 | -0.007 | 0.920 |
| cg06810647 | -0.007 | 0.921 |
| cg22947000 | 0.006 | 0.933 |
| cg17324128 | -0.005 | 0.940 |
| cg19706682 | 0.005 | 0.943 |
| cg10865119 | -0.004 | 0.950 |
| cg02331561 | -0.004 | 0.952 |
| cg05294243 | -0.004 | 0.952 |
| cg10920957 | 0.004 | 0.954 |
| cg19514928 | 0.004 | 0.954 |
| cg08370996 | -0.004 | 0.956 |
| cg13216057 | 0.004 | 0.958 |
| cg07730301 | -0.004 | 0.959 |
| cg04999691 | 0.003 | 0.960 |
| cg08331960 | -0.003 | 0.961 |
| cg11653266 | 0.003 | 0.963 |
| cg22197830 | -0.003 | 0.966 |
| cg19761273 | 0.003 | 0.968 |
| cg15703512 | -0.003 | 0.970 |
| cg06462291 | -0.002 | 0.972 |
| cg26614073 | 0.002 | 0.974 |
| cg02217159 | 0.002 | 0.975 |
| cg01584473 | -0.002 | 0.978 |
| cg13460409 | 0.002 | 0.978 |
| cg20295671 | 0.002 | 0.979 |
| cg06993413 | -0.002 | 0.980 |
| cg00864867 | -0.002 | 0.982 |
| cg03760483 | -0.001 | 0.984 |
| cg07455279 | 0.001 | 0.984 |
| cg20100381 | 0.001 | 0.986 |
| cg17729667 | 0.001 | 0.992 |
| cg18573383 | 0.001 | 0.996 |
| cg21096399 | 0.001 | 0.996 |
| cg24058132 | 0.001 | 0.997 |
| cg10376763 | 0.001 | 0.999 |

| Table S3 The composite CpGs of Horvath age related to changes in liver fat content by univariate correlation analysis | | | | | | |
| --- | --- | --- | --- | --- | --- | --- |
| **CpG** | **Chromosome** | **Position** | **Gene** | **Metabolic function** | **Correlation coefficient** | ***P* value** |
| cg01353448 | 7 | 31726912 | ***C7orf16*** | **Restricts food intake^1^** | 0.145 | 0.032 |
| cg01485645 | 17 | 36862199 | ***MLLT6*** | **Modulates sodium homeostasis and blood pressue^2^; abnormal liver morphology^3^** | 0.165 | 0.014 |
| cg01560871 | 10 | 72545424 | *C10orf27* | Modulates thymic stromal cell proliferation and thymus function^4^ | 0.138 | 0.041 |
| cg03103192 | 4 | 52917271 | *SPATA18* | Unknown | 0.142 | 0.034 |
| cg03947362 | 2 | 200820154 | ***C2orf69;C2orf47*** | **Modulates mitochondrial function and regulates liver glycogen and lipid storage^5^** | 0.134 | 0.047 |
| cg08090772 | 8 | 67344640 | ***ADHFE1*** | Differentiation-dependent expression in adipogenesis^6^ | 0.135 | 0.046 |
| cg08251036 | 2 | 135008923 | ***MGAT5*** | **Increases body weight and hepatic lipogenesis^7^** | -0.146 | 0.030 |
| cg14308452 | 19 | 5784184 | *PRR22* | Unknown | 0.168 | 0.013 |
| cg16241714 | 8 | 48650511 | ***CEBPD*** | **Promotes adipogenesis^8^** | 0.152 | 0.024 |
| cg18139769 | 7 | 94286955 | ***SGCE;PEG10*** | **Reduces liver polyunsaturated fatty acids and promotes liver lipogenesis^9^** | -0.137 | 0.042 |
| cg20305610 | 4 | 95373302 | ***PDLIM5*** | **Associates with type 2 diabetes and hypertension^10^** | 0.154 | 0.022 |
| cg22432269 | 15 | 22892697 | *CYFIP1* | Unknown | -0.200 | 0.003 |
| cg23092072 | 4 | 87927706 | ***AFF1*** | **Inhibits adipogenic differentiation^11^** | -0.161 | 0.017 |
| cg24450312 | 1 | 206681158 | *RASSF5* | **Associates with obesity and type 2 diabetes^12^** | 0.145 | 0.032 |
| cg25070637 | 8 | 97505868 | ***SDC2*** | **Influences circulating triglyceride level^13^; Increases during transformation from fat cell to myofibroblasts^14^** | -0.151 | 0.024 |

* Functions associated with liver fat are bolded.

1. Caglar C, Friedman J. Restriction of food intake by PPP1R17-expressing neurons in the DMH. Proc Natl Acad Sci U S A. 2021;118(13):e2100194118.

2. Chen L, Wu H, Pochynyuk OM, Reisenauer MR, Zhang Z, Huang L, et al. Af17 deficiency increases sodium excretion and decreases blood pressure. J Am Soc Nephrol. 2011;22(6):1076-86.

3. <http://www.informatics.jax.org/marker/phenotypes/MGI:1935145>

4. <https://www.uniprot.org/uniprotkb/Q96M53/entry>

5. Lausberg E, Gießelmann S, Dewulf JP, et al. C2orf69 mutations disrupt mitochondrial function and cause a multisystem human disorder with recurring autoinflammation. J Clin Invest. 2021;131(12):e143078.

6. Kim JY, Tillison KS, Zhou S, Lee JH, Smas CM. Differentiation-dependent expression of Adhfe1 in adipogenesis. Arch Biochem Biophys. 2007;464(1):100-11.

7. Ryczko MC, Pawling J, Chen R, Abdel Rahman AM, Yau K, et al. Metabolic Reprogramming by Hexosamine Biosynthetic and Golgi N-Glycan Branching Pathways. Sci Rep. 2016;6:23043.

8. Choy L, Derynck R. Transforming growth factor-beta inhibits adipocyte differentiation by Smad3 interacting with CCAAT/enhancer-binding protein (C/EBP) and repressing C/EBP transactivation function. J Biol Chem. 2003;278(11):9609-19.

9. Arendt BM, Comelli EM, Ma DW, et al. Altered hepatic gene expression in nonalcoholic fatty liver disease is associated with lower hepatic n-3 and n-6 polyunsaturated fatty acids. Hepatology. 2015;61(5):1565-78.

10. Owusu D, Pan Y, Xie C, Harirforoosh S, Wang KS. Polymorphisms in PDLIM5 gene are associated with alcohol dependence, type 2 diabetes, and hypertension. J Psychiatr Res. 2017;84:27-34.

11. Chen Y, Wang Y, Lin W, Sheng R, Wu Y, Xu R, et al. AFF1 inhibits adipogenic differentiation via targeting TGM2 transcription. Cell Prolif. 2020;53(6):e12831.

12. Kirchner H, Sinha I, Gao H, et al. Altered DNA methylation of glycolytic and lipogenic genes in liver from obese and type 2 diabetic patients. Mol Metab. 2016;5(3):171-183.

13. <http://www.informatics.jax.org/marker/phenotypes/MGI:1349165>

14. Weiner OH, Zoremba M, Gressner AM. Gene expression of syndecans and betaglycan in isolated rat liver cells. Cell Tissue Res. 1996;285(1):11-6.

| Table S4 Comparison of the diagnostic performance of the scores for predicting NAFLD among participants from both cohorts (n=291) | | | | | | | | | | | | | | | | |
| --- | --- | --- | --- | --- | --- | --- | --- | --- | --- | --- | --- | --- | --- | --- | --- | --- |
|  | AUROC (95%CI) | Rule-out zone | | | | | | | indeterminate | Rule-in zone | | | | | | |
|  |  | Cut-offs | n (%) | Sensitivity | | Specificity | NPV | |  | Cut-offs | n (%) | | Sensitivity | Specificity | | PPV |
| NAFLD Clin Score | 0.70(0.64-0.77) | -1.74 | 81(27.8%) | 90.0% | | 33.8% | 89.7% | | 164(56.4%) | -0.63 | 46(15.8%) | | 32.4% | 90.0% | | 48.8% |
| NAFLD Methyl Score | 0.77(0.70-0.83) | -1.77 | 107(36.8%) | 90.0% | | 46.1% | 92.2% | | 131(45.0%) | -0.41 | 53(18.2%) | | 42.6% | 90.0% | | 54.9% |
| NAFLD Clin Score+Age acceleration | 0.76(0.70-0.81) | -1.55 | 123(42.3%) | 90.0% | | 53.4% | 93.3% | | 128(44.0%) | -0.35 | 40(13.7%) | | 26.5% | 90.0% | | 44.7% |
| NAFLD ClinMethyl Score | 0.81(0.76-0.87) | -1.60 | 140(48.1%) | 90.0% | | 59.8% | 94.6% | | 96 (33.0%) | -0.33 | 55(18.9%) | | 45.6% | 90.0% | | 57.4% |
|  |  |  | | |  | |  |  | | | |  | | |  | |


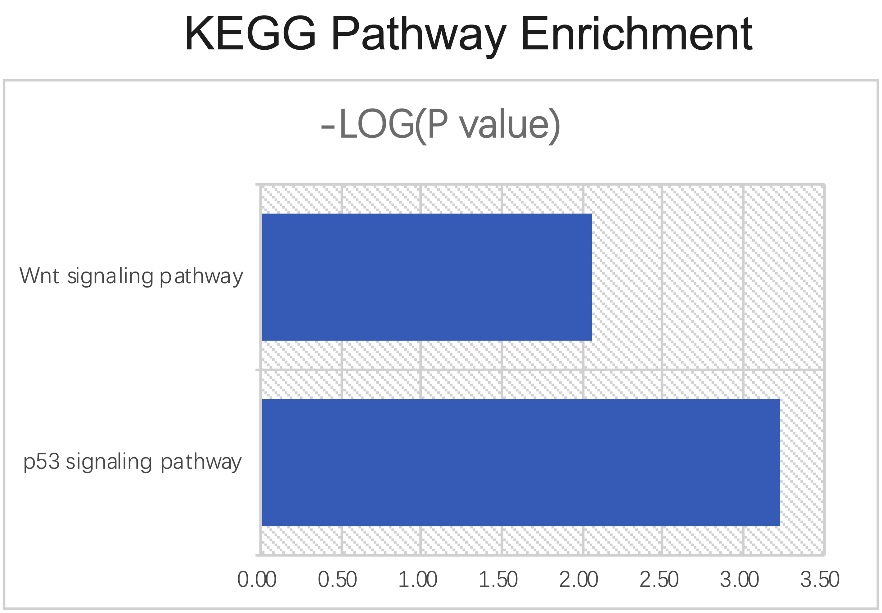


Figure S1 Pathway analysis indicated that the genes where the top CpGs located were enriched in wnt signaling pathway and p53 signaling pathway.


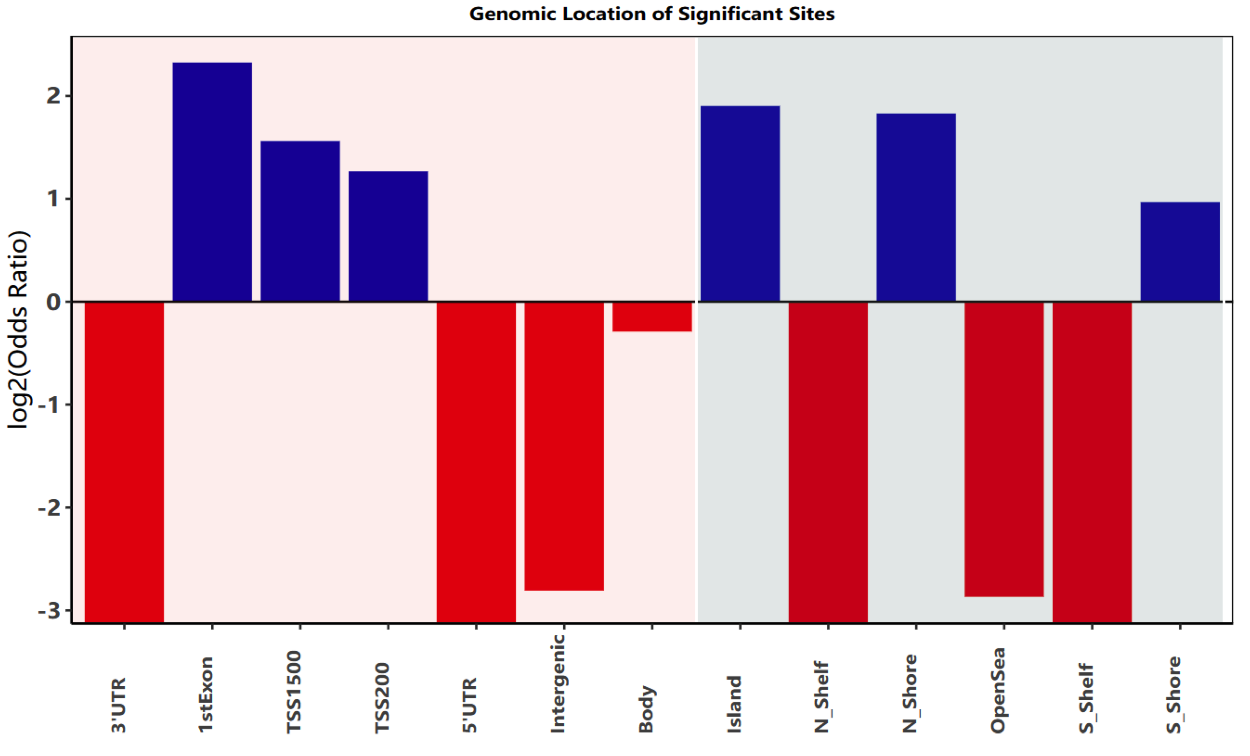


Figure S2 Genomic location of CpG sites that were significantly associated with longitudinal changes in liver fat content.
